# *SoxNeuro* orchestrates central nervous system specification and differentiation in *Drosophila* and is only partially redundant with *Dichaete*

**DOI:** 10.1186/gb-2014-15-5-r74

**Published:** 2014-05-30

**Authors:** Enrico Ferrero, Bettina Fischer, Steven Russell

**Affiliations:** 1 Department of Genetics, University of Cambridge, Downing Street, CB2 3EH Cambridge, UK; 2 Cambridge Systems Biology Centre, University of Cambridge, Tennis Court Road, CB2 1QR Cambridge, UK

## Abstract

**Background:**

Sox proteins encompass an evolutionarily conserved family of transcription factors with critical roles in animal development and stem cell biology. In common with vertebrates, the *Drosophila* group B proteins SoxNeuro and Dichaete are involved in central nervous system development, where they play both similar and unique roles in gene regulation. Sox genes show extensive functional redundancy across metazoans, but the molecular basis underpinning functional compensation mechanisms at the genomic level are currently unknown.

**Results:**

Using a combination of genome-wide binding analysis and gene expression profiling, we show that SoxNeuro directs embryonic neural development from the early specification of neuroblasts through to the terminal differentiation of neurons and glia. To address the issue of functional redundancy and compensation at a genomic level, we compare SoxNeuro and Dichaete binding, identifying common and independent binding events in wild-type conditions, as well as instances of compensation and loss of binding in mutant backgrounds.

**Conclusions:**

We find that early aspects of group B Sox functions in the central nervous system, such as stem cell maintenance and dorsoventral patterning, are highly conserved. However, in contrast to vertebrates, we find that *Drosophila* group B1 proteins also play prominent roles during later aspects of neural morphogenesis. Our analysis of the functional relationship between SoxNeuro and Dichaete uncovers evidence for redundant and independent functions for each protein, along with unexpected examples of compensation and interdependency, thus providing new insights into the general issue of transcription factor functional redundancy.

## Background

The evolution of multicellular organisms is, to a large extent, driven by an increase in the complexity of gene regulatory networks [1], both at the level of *cis*-regulatory elements [2] and of transcription factor (TF) diversity [3]. In metazoans, many TFs have arisen through local tandem or whole genome duplications followed by neofunctionalisation, a process leading to the generation of new regulatory networks or the modification of existing ones. These processes generate developmental diversity and ultimately species evolution. Interestingly, some duplicated genes can maintain redundant functions over very substantial periods of time [4], an observation that appears to be counterintuitive from the perspective of natural selection. In general, it is expected that duplicated genes either diverge to generate new functions or one of the paralogs is lost through the accumulation of inactivating mutations [5]. It has been suggested that redundancy may be maintained when duplicates have multiple functions, both common and unique, that would otherwise be eliminated by deleterious mutations [4]. While such models account for the maintenance of closely related coding sequences in the genome, they do not explain why redundant copies do not always diverge to adopt different expression domains [5]. In some cases, it is possible that maintaining partially redundant genes with similar expression patterns may contribute to network robustness [6]; however, we lack sufficient data on the genome-wide activities of paralogous TFs to make reliable inferences about the molecular mechanisms underlying redundancy.

The Hox family of TFs, which share a conserved organisation and function during embryonic segmentation, exemplifies the expected evolutionary trajectory of duplication events [7], with paralogous genes showing divergent expression domains and strong phenotypes when individually deleted, although analysis of double mutants suggests a limited degree of functional redundancy in some cases [8]. In contrast, Sox (SRY-related high-mobility-group box) genes, another family of metazoan TFs that have arisen through gene duplications [9-11], exhibit a much higher degree of functional redundancy, with closely related genes often widely coexpressed and able to substantially compensate for each other's loss [12-17]. The reasons why some TF families have functionally diverged while others have maintained considerable redundancy is a fascinating unanswered question.

Sox proteins have established roles in transcriptional regulation and may also play an architectural role in chromatin organisation [18,19]. The 20 Sox genes in vertebrates are subdivided into 8 groups (A to H), most of which contain multiple paralogs. Group B genes are of particular interest from an evolutionary perspective, providing examples of both neofunctionalisation and redundancy. This group is divided into two further subgroups, B1 (*Sox1*, *Sox2*, and *Sox3*) and B2 (*Sox14* and *Sox21*) [10], both playing important roles during vertebrate neurogenesis. SoxB1 proteins primarily act as transcriptional activators, in particular regulating the maintenance of neural stem cell (NSC) self-renewal, while SoxB2 proteins mainly function as transcriptional repressors, promoting the differentiation of neural precursors into mature neurons [20-22]. In most vertebrates, the three B1 proteins are extensively coexpressed in the developing central nervous system (CNS) and single gene mutants or knockdowns show only mild embryonic CNS phenotypes [23-25]. In zebrafish, where four group B1 genes are coexpressed in the CNS, only knockdown of all four elicits a severe CNS phenotype, with single, double and even triple mutant combinations showing substantial CNS development [26]. On the one hand, the evolution of diversified roles for B1 and B2 proteins illustrates neofunctionalisation, but on the other, the extensive coexpression of B1 proteins in the early CNS across the vertebrates represents a prime example of conserved functional redundancy.

The *Drosophila melanogaster* genome encodes four group B genes (*SoxNeuro* (*SoxN*), *Dichaete* (*D*), *Sox21a*, *Sox21b*) [27]. While there is still some uncertainty regarding the B1 and B2 subdivision in insects, with different views on their grouping and evolution proposed [10,11,28,29], at a functional level SoxB factors appear to be functionally conserved across the metazoa, with mammalian SoxB1 proteins able to rescue *Drosophila* mutations [30,31]. While the functions of *Sox21a* and *Sox21b* are currently unknown [27], *SoxN* and *Dichaete* have prominent roles in CNS development and exhibit extensive functional redundancy [32,33]. Both genes are dynamically expressed in partially overlapping domains of the embryonic CNS [34-38] and double mutants display far more severe CNS phenotypes than either single mutant. Along with redundant functions, each gene has unique expression domains and, in some circumstances, the two TFs also appear to have opposite functions in gene regulation [32,33,38,39]. The conservation in group B Sox function, combined with the evidence that individual members can have both unique and redundant functions, makes *Drosophila* an attractive system for studying redundancy between paralogous TFs.

*SoxN* and *Dichaete* are involved in many of the pathways controlling neural specification in *Drosophila* and there are striking similarities to the roles played by vertebrate group B Sox proteins that suggest an underlying conservation [20]. For example, vertebrate B1 proteins have critical roles in the maintenance of NSCs [40,41] and both fly genes are required for the correct establishment of neuroblasts (NBs), the fly equivalent of vertebrate NSCs [33,39], with Dichaete known to be involved in maintaining embryonic and larval NBs in a self-renewing state [42]. At the molecular level, Dichaete interacts with the POU protein Ventral veins lacking (Vvl) to regulate gene expression in the CNS midline [43], a role reminiscent of the Sox2-Oct4 interaction required for stem cell maintenance in mammals [44-46]. Similarly, a set of homeodomain proteins are critical for patterning the dorsoventral (DV) axis of the CNS in both vertebrates and *Drosophila*, where they are coexpressed and interact with SoxB proteins [20,33,38,47]. Despite these known functional and molecular similarities, how widely group B Sox functions are conserved between invertebrates and vertebrates remains to be determined.

Genome-wide studies analysing global gene expression changes or patterns of genomic binding can provide significant insights into the function of TFs. Recently, a genome-wide study in mouse neural cells highlighted extensive overlap between Sox2 and Sox3 binding, supporting the view that SoxB1 proteins are functionally redundant [48]. In *Drosophila*, genome-wide analysis of Dichaete implicates it in the regulation of hundreds of genes in the CNS [39,49]. Here, we focus on a genomic analysis of SoxN, identifying hundreds of putative direct SoxN target genes. We then tackle the issues of redundancy and compensation between SoxN and Dichaete by generating binding profiles for both factors in wild-type and mutant backgrounds. We identify instances of redundancy and compensation at the molecular level, as well as other changes in the binding profiles indicative of interdependency between the two factors. Our comparative analysis provides the first molecular view of functional redundancy and compensation between paralogous TFs at a genome-wide scale, and provides new insights into the functional conservation of group B Sox genes in animals.

## Results

### Gene expression changes in *SoxN* mutants

**Figure 1:**
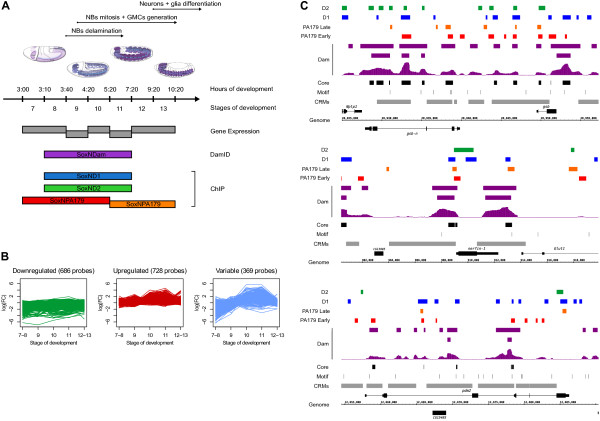
**SoxN functional studies. (A)** Overview of the SoxN datasets generated. Each coloured box below the time line represents a single gene expression, DNA adenine methyltransferase identification (DamID) or chromatin immunoprecipitation (ChIP) experiment performed at the indicated time ranges. Above the time line, major events in neural development are indicated and illustrated with FlyBase images adapted from Volker Hartenstein, *Atlas of* Drosophila *Development*, Cold Spring Harbor Laboratory Press, 1993. **(B)** Partitioning of genes differentially expressed in *SoxN* mutants. Probes corresponding to differentially expressed genes were divided into three groups (downregulated, upregulated and variable) according to their expression trend over time. **(C)** Genomic profiles of SoxN binding. Window scores and binding intervals at a false discovery rate (FDR) of 1% and FDR of 25% are displayed for the SoxNDam dataset (purple), and binding intervals at FDR 25% are shown for the SoxNPA179 Early (red), SoxPA179 Late (orange), SoxND1 (blue) and SoxND2 (green) ChIP datasets. SoxN core binding intervals are displayed in black, matches to the SoxN binding motif as thin bars and the locations of know *cis*-regulatory modules (CRMs) as grey bars. SoxN binding in the *gsb-n* and *gsb* (top panel), *nerfin-1* (middle panel) and *pdm2* (bottom panel) regions are displayed.

We recently performed genomic analyses of the role of Dichaete in the embryonic nervous system that identified hundreds of target genes with diverse roles in CNS development [39,49]. Here, we determine the functions of SoxN during embryonic development by profiling temporal changes in the transcriptome of *SoxN* mutants and by mapping the genome-wide binding of SoxN. To capture expression changes and binding events relevant to neural development, from the specification of NBs through to the terminal differentiation of neurons and glial cells, experiments were performed at specific time points encompassing stages 7 to 13 of embryogenesis (Figure 1A).

We extracted RNA from *SoxN* hemizygous null mutants and compared it with RNA from their heterozygous siblings via biologically replicated hybridisations to long oligonucleotide microarrays, across five developmental time points. After normalisation and statistical thresholding of these data, a total of 1,783 probes, corresponding to 1,665 genes, were differentially expressed across the time course (Table S1A-C in Additional file 1). At each time point, a score of -1, 0 or 1 was attributed to all genes showing a significant differential expression according to the corrected *P*-value associated with a moderated F-statistic and the direction of the expression change at each time point. This led to the identification of genes up- and downregulated in the mutants across the whole time course, as well as a third set of genes more variably expressed across the time course (Figure 1B). The enrichment in Gene Ontology biological process (GO:BP) terms in these three groups showed a marked difference (Figure S1A in Additional file 2 and Table S1D-I in Additional file 1). The 647 downregulated genes were the most relevant from a neural development perspective, being enriched in transcriptional regulation and specific terms related to early and late CNS development. This indicates that many of the genes directly or indirectly activated by SoxN are involved in controlling gene expression during neural development, in processes ranging from NB fate commitment through to neuronal development and differentiation. Conversely, while the list of 679 upregulated genes contained a few examples of genes known to play a role in the CNS, the list was enriched for very few nervous system GO terms but overrepresented for stress response terms, suggesting that at least some of the upregulated genes may represent a reaction to development in the absence of transcriptional regulators such as SoxN and its downstream targets. Finally, the set of variable genes, most of which were downregulated until stage 9, showed increased expression during stages 10 to 11 and returned to basal levels at the latest stages analysed, showed little significant GO enrichment, although we noticed several genes with known roles in CNS development (for example, *beat-Ia*, *Fas3*, *frac*, *Kr-h1*, *lbl*, *Lim3*). Overall, these data suggest that SoxN mainly functions in the nervous system as a transcriptional activator to promote the expression of both transcriptional regulators and effectors involved at all stages of neural development but may also act to repress some genes with CNS functions as well as more generic biological functions.

### A genome-wide view of SoxN binding

To map high confidence SoxN binding intervals across the genome we employed two complementary approaches, DNA adenine methyltransferase identification (DamID) and chromatin immunoprecipitation (ChIP), using genome-wide tiling arrays. We first used DamID to generate a reference profile of SoxN binding across stages 8 to 11 of embryogenesis (SoxNDam). Next, to provide independent validation of the DamID binding, we also produced a set of four ChIP datasets, employing three different antisera. Two of the antisera (SoxND1 and SoxND2) were used to generate SoxN ChIP profiles across the same developmental stages as the DamID experiment. We also generated a new affinity purified antiserum (SoxNPA179), showing consistent SoxN expression by whole-mount immunohistochemistry, and used this to create two further datasets (SoxNPA179 Early, stages 7 to 10, and SoxNPA179 Late, stages 11 to 13).

All of the DamID and ChIP data were similarly processed and bound regions were identified according to a false discovery rate (FDR) model (Table 1A). Based on the smoothed window score profiles and the number of binding intervals detected, we focused on stringent FDR 1% data from the SoxNDam and the SoxND1 and SoxND2 ChIP experiments. In the case of the SoxNPA179 ChIP datasets, we reasoned that the narrower time windows employed could restrict the identification of comparable binding intervals and we selected the FDR 5% datasets for further analysis (Table 1B). We compared the binding intervals and associated genes from DamID and ChIP assays (Figure 1C; Figure S1B,C in Additional file 2) and found a general concordance between the datasets. We then combined the five datasets to generate a core set of SoxN binding intervals that we used for further analysis. Since we only selected binding intervals with supporting DamID and ChIP evidence, this is a conservative approach and it is likely that SoxN interacts with a larger fraction of the genome than we report here.

**Table 1:** SoxN binding datasets, intervals and genes

**A**	**FDR 1%**	**FDR 5%**	**FDR 10%**	**FDR 25%**
SoxNDam	6,518	11,133	14,223	19,186
SoxND1	5,830	11,988	16,786	27,272
SoxND2	5,599	7,904	9,650	13,424
SoxNPA179 Early	3,145	6,335	9,001	15,818
SoxNPA179 Late	1,556	3,502	5,348	10,437
**B**		**Intervals**	**Genes**	
SoxNDam FDR 1%		6,518	3,557	
SoxND1 FDR 1%		5,830	4,212	
SoxND2 FDR 1%		5,599	4,073	
SoxNPA179 Early FDR 5%		6,335	4,529	
SoxNPA179 Late FDR 5%		3,502	2,652	

**(A)** Number of intervals retrieved by the peak calling algorithm for the five binding datasets at different FDRs. **(B)** Number of genes associated with each high confidence binding dataset.

**Figure 2:**
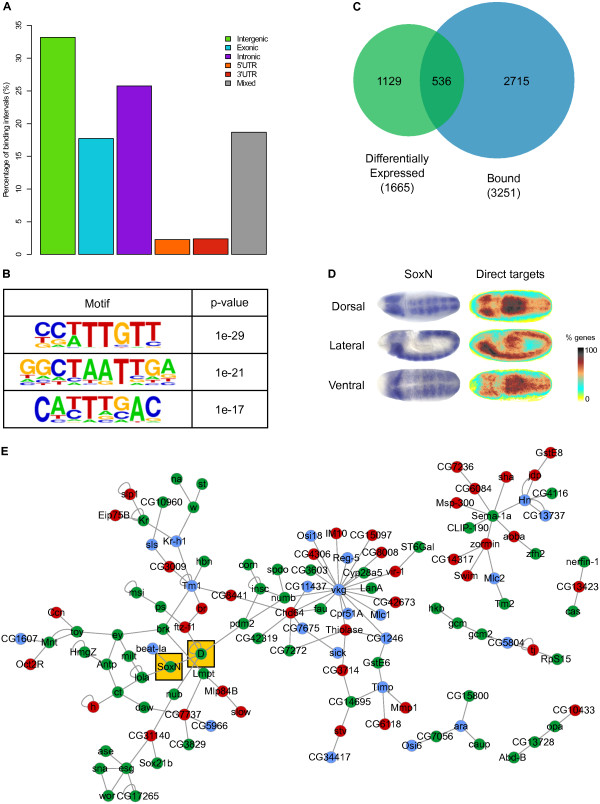
**Features of SoxN binding and SoxN direct targets. (A)** Barplot representing genomic features hit by SoxN binding intervals. 'Mixed' indicates intervals hitting more than a single feature in different genes. **(B)***De novo* motifs discovered in the SoxN core dataset. The top three motifs found and their associated *P*-values are reported. **(C)** Proportional Venn diagram showing the overlap between genes differentially expressed in *SoxN* mutants (green, left) and genes bound by SoxN (blue, right). **(D)** Dorsal, lateral and ventral views of SoxN expression in a stage 9-10 embryo (left, images from the Berkeley *Drosophila* Genome Project) and false-colour heatmaps representing the average expression pattern of SoxN direct targets at the same developmental stages. **(E)** Network showing known interactions between SoxN direct targets obtained by superimposing the list of SoxN direct targets with a network created from the DroID database. Genes are colour-coded according to their expression trend during the time-course (green, downregulated; red, upregulated; light blue, variable). SoxN and Dichaete are highlighted with yellow boxes.

These combined DamID and ChIP data identified 5,482 SoxN binding intervals associated with 3,251 genes, enriched for GO:BP terms relating to general and nervous system development, as well as RNA transcription and regulation (Table S2A-C in Additional file 3). To support the reliability of our analysis, we assessed the overlap between our SoxN-bound genes and those identified in a previously published small-scale ChIP analysis of SoxN binding [50]. Of 26 SoxN-bound genes identified in this study, 18 are present in our core binding interval set, a further 4 showed evidence of SoxN binding but below our threshold and only 4 were negative in our assays. Looking at the general properties of SoxN binding intervals, we found they are often in close proximity to transcription start sites (TSSs; Figure S2A in Additional file 4); however, there does not seem to be preferential binding of SoxN upstream of the TSS, since the fraction of intervals mapping upstream (47.6%) or downstream (52.3%) is comparable. Interestingly, Sox2 binding in the vicinity of TSS has also been reported [51]. We used the midpoint of each binding interval to assess the genomic features associated with SoxN binding and found a high proportion mapping to genic (66.8%) rather than intergenic (33.2%) regions. Notably, within genes, we found that introns (25.8%) were more targeted than exons (17.7%) and that UTRs accounted for only 4.7% of intervals (Figure 2A). The binding intervals were divided into the three main categories intergenic, intronic and exonic, and gene lists were generated for each set (Table S2D-I in Additional file 3). Remarkably, The GO:BP enrichment computed for each of the resulting gene lists showed considerable differences (Figure S2B in Additional file 4; Table S2J-L in Additional file 3). Intergenic hits were highly enriched in processes related to the regulation of transcription and gene expression, while intronic hits had a clear developmental signature containing terms related to neurogenesis and morphogenesis. The level of enrichment found for exonic hits was substantially lower than those observed for the two other categories and only featured generic GO:BP terms.

We examined the sequence composition of SoxN binding intervals and noticed a marked increase in the average GC content at the centre of the intervals compared to the flanking 10 kb (Figure S2C in Additional file 4). The GC content profile mirrored the average phastCons score distribution calculated from multiple genome alignments of *D. melanogaster* with 14 other insect species (Figure S2D in Additional file 4), showing that core SoxN binding intervals are well conserved. Sox domains bind to a conserved DNA motif (5'-WTTGWW-3') [52,53], and scanning the core binding intervals with positional weight matrices from different species revealed high scoring matches to known Sox motifs (Figure S2E in Additional file 4). Similarly, performing a *de novo* motif discovery search identified a top-scoring motif closely matching the Sox consensus (Figure 2B). Similar, but not identical, motifs are reported for Dichaete [54,55]. We mapped high scoring matches (*P* < 1E-4) to the new SoxN motif across the genome [56] and identified over 43,000 matches, displayed as tracks in Figure 1C and subsequent binding profile figures, that show a good correspondence with the binding intervals we selected. The second and third highest scoring motifs identified in the *de novo* search are similar to homeobox binding sites. In particular, we note that motif 2 is very similar to those reported for Dr and Ind [54], while motif 3 closely resembles that of Vnd [57], three proteins playing key roles in the specification of neural identity across the DV axis. Thus, our analysis reveals a core set of well-conserved SoxN binding intervals, enriched for a novel SoxN binding motif, along with motifs associated with other TFs involved in *Drosophila* CNS development.

We compared our core SoxN binding intervals with binding intervals and enriched chromatin domains reported by the Berkeley Drosophila Transcription Network Project (BDTNP) [58,59] and the Model Organism Encyclopedia of DNA Elements (modENCODE) [60,61]. We found highly significant (z-score > 200) overlaps between binding intervals for SoxN and several TFs, including a number known to be involved in aspects of embryonic nervous system development (Hb, Kr, Dichaete, Med, Sens and Da). Many of the genes for these TFs (Hb, Kr, Dichaete and Med) contain SoxN binding intervals, suggesting that SoxN may regulate as well as interact with them during CNS development. As expected, the profile of SoxN overlaps is very similar to those observed with other TFs involved in CNS development (Hkb, Kr, Ubx and Zfh1; Figure S3A in Additional file 5). We also identified significant overlaps between SoxN binding and some histone-modifying proteins (particularly histone acetyltransferases and deacetylases) as well as domains enriched for several histone modifications. The majority of the histone modifications overlapping with SoxN binding are associated with active chromatin. However, we also found an association with histone marks normally associated with transcriptional silencing or repression, suggesting that SoxN may also act as a transcriptional repressor. Alternatively, this may highlight bivalent areas containing marks for both activation and repression that are poised for transcription [62], or it may simply reflect the fact that across the embryo some genes are repressed in particular cell lineages and active in others. As with the TF overlap, the pattern observed with SoxN is very similar to those observed with other nervous system regulators (Figure S3B in Additional file 5).

To link SoxN binding with mapped *cis*-regulatory modules (CRMs) in the *Drosophila* genome, we compared the core binding intervals with enhancer regions defined by REDFly (1,864 CRMs from 500 genes) [63] and FlyLight (7,113 CRMs from 970 genes) [64]. We found SoxN binding overlapping with 1,511 of 8,959 (17%) unique CRMs defined by both databases, including 704 out of the 4,724 (15%) FlyLight enhancers reported to show CNS expression (Table 2). Taken together, these observations support the general conclusion that SoxN acts as a transcriptional activator, interacting with other TFs at known CRMs, to control expression of a set of genes essential for CNS development.

**Table 2:** **Overlaps between SoxNeuro core intervals and known *cis*-regulatory modules**

	**CRMs**	**CRMs overlapping with SoxN core intervals**	**Genes**	**Genes overlapping with SoxN core intervals**	**Genes in SoxN core**
REDfly	1,864	492 (26.4%)	500	135 (27.0%)	218 (43.6%)
FlyLight	7,113	1,023 (14.4%)	970	342 (35.3%)	364 (37.5%)
FlyLight CNS	4,724	704 (14.9%)	780	267 (34.2%)	307 (39.4%)
All (REDfly + FlyLight)	8,959	1,511 (16.9%)	1,302	418 (32.1%)	477 (36.6%)

Number and percentages of CRMs and associated genes from REDfly and/or FlyLight displaying overlap with SoxNeuro core intervals. 'FlyLight CNS' refers to FlyLight CRMs reportedly driving expression in the CNS. 'All' refers to the total number of unique CRMs in the REDfly and FlyLight sets combined together.

### Identification of SoxN direct targets

To uncover a high confidence set of SoxN target genes, we intersected the differential expression data (1,665 genes) and core SoxN binding intervals (3,251), identifying 536 genes that we assigned as direct SoxN targets. We added a further 7 genes that were not identified as SoxN bound because of the computational approach we used to assign intervals to genes, resulting in 543 targets (Figure 2C; Table S3A in Additional file 6). Of these, 199 genes were consistently downregulated, 213 upregulated and 131 variable in the microarray time course (Table S3B-D in Additional file 6). We emphasise this is a conservative estimate since our stringent selection criteria for binding and differential expression are likely to exclude many *bona fide* binding events and small, but functionally relevant, changes in gene expression. In addition, loss of SoxN binding at some genomic locations is likely to be rescued by Dichaete activity (see below). Despite these caveats, we found that approximately a third of genes with significant expression changes in *SoxN* mutant embryos were also bound by SoxN, and that over 15% of SoxN-bound genes showed expression changes at our significance threshold. As expected, we found that the GO:BP enrichment was similar to that of the two original datasets, with development and transcription-related terms overrepresented (Table S3E in Additional file 6). Enrichment of more specific terms associated with NB specification and fate commitment, and a range of terms relating to the development of glia, neurons and their projections was also found.

We examined the embryonic expression patterns of the SoxN target genes using genome-wide expression maps [65], and found that the average expression of the target genes closely matches that of SoxN CNS expression, supporting the reliability of our dataset (Figure 2D). Using the DroID database [66], we rendered a network featuring all known high confidence *Drosophila* genetic and protein-protein interactions, and superimposed our list of SoxN targets onto this. All modules with more than two nodes were selected to retrieve the most significant known interactions between SoxN direct targets (Figure 2E). The resulting subnetworks are highly interconnected and contain many proteins involved in specific aspects of nervous system development such as asymmetric NB division (Insc, Numb, Spdo, Sna, Wor and Esg), gliogenesis (Hkb, Gcm and Gcm2) and eye development (Ey and Toy), as well as most of the TFs involved in the temporal progression of NB identity (Cas, D, Kr, Nub and Pdm2). We also identified proteins specifically involved in the development of neuronal projections (Ct, Daw, Nerfin-1 and Sema-1a), and a set of homeodomain-containing proteins (Abd-B, Antp, Ara, Caup and Zfh2) with various roles in the CNS.

**Figure 3:**
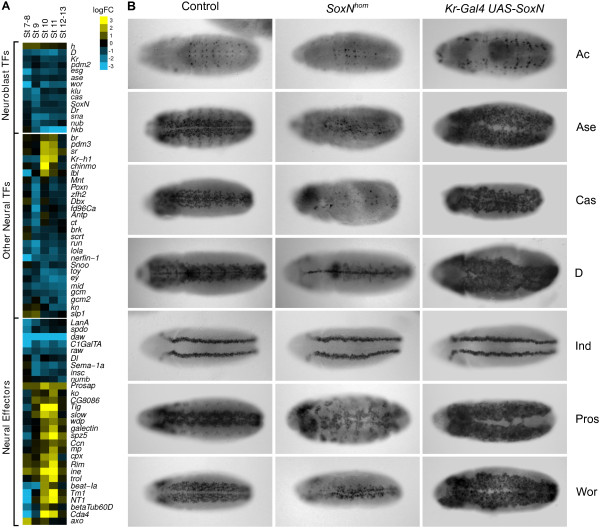
**Validation of SoxN direct targets. (A)** Selected genes from a hierarchical clustering of expression trends highlighting three functional groups of SoxN direct targets. logFC = log_2_ fold change. **(B)** Immunohistochemical stainings of a selection of SoxN direct targets identified in this study in control embryos (left), *SoxN* homozygous mutants (middle) and *Kr-Gal4 UAS-SoxN* embryos (right). All embryos are shown as ventral views with anterior to the left. Ind, showing normal expression in mutant and transgenic embryos, is provided as a negative control.

Our analysis indicated that SoxN directly regulates a large group of TFs and effectors with a range of diverse functions in CNS development as illustrated with a selection of genes taken from a clustering analysis (Figure 3A). To confirm this, 29 of the most functionally relevant genes, including 19 from the network described above, were selected for validation by immunohistochemistry or *in situ* hybridization. These included proneural genes, TFs controlling NB divisions and identity, as well as TFs involved in aspects of glial or neuronal differentiation such as axon fasciculation. Strikingly, the expression of all of these was disrupted in *SoxN* mutants, in many cases very severely (Figure 3B; Additional file 7). In particular, we frequently observed reduced and/or altered expression patterns in the most lateral domains of the neuroectoderm, where Dichaete is not expressed and therefore unable to functionally compensate for the loss of SoxN. We also examined the expression of a selection of these targets in embryos ectopically expressing SoxN via a *Kr*-Gal4 driver (Figure 3B). We found that Ac, Ase, Cas, Dichaete, Pros and Wor expression was severely altered in SoxN misexpressing embryos, with increased and ectopic expression in the lateral domains of the neuroectoderm, supporting the view that our proposed targets are under direct SoxN transcriptional control. Analysis of genes not expected to be affected by loss or gain of *SoxN* (*ind* in the medial column of the neuroectoderm (Figure 3B), and *sim* in the midline (Figure S4A in Additional file 7)) indicate that the expression phenotypes we observe are not due to a general disruption in the organisation of the CNS. Overall, we have identified key roles for SoxN in all aspects of embryonic CNS development, and showed that it regulates sets of TFs and effectors involved in processes ranging from the earliest events in neural identity specification to the terminal differentiation of neurons and glia.

To relate the activity of SoxN to its mammalian orthologues, we compared the set of SoxN-bound genes with those identified as Sox2 or Sox11 targets in mouse. Bergsland and colleagues [48] identified 1,388 regions bound by Sox2 in neural precursor cells that correspond to 1,100 genes. We mapped these genes to their *Drosophila* orthologues and found that 443 are conserved in our set of SoxN targets (Table S4A in Additional file 8). In other words, more than 40% of Sox2-bound genes are also bound by SoxN, but this core of conserved targets represents only approximately 13.5% of SoxN-bound genes, suggesting that SoxN has more diversified functions than Sox2 in the CNS. The list of shared targets is, as expected, enriched for TFs and effectors with roles in CNS development (Table S4B in Additional file 8), including the DV patterning homeobox genes *Dr* and *vnd*, proneural genes, bHLH genes in the Enhancer of split complex and many other transcriptional regulators whose absence is known to cause CNS phenotypes in both organisms. In the case of Sox11, a group C Sox protein involved in neural differentiation, we found a much larger overlap. Over a third of the SoxN bound genes (34%, 1,092 genes) have mouse orthologues bound by Sox11 in neural precursors or differentiating neural cells (Table S4C in Additional file 8), including TFs and effectors with roles in both early neural specification and neuron differentiation (Table S4D in Additional file 8). We also identified 722 genes bound by SoxN and Sox11 but not Sox2 (Table S4E in Additional file 8), which are enriched for terms related to neuronal projection development and morphogenesis (Table S4F in Additional file 8). Together, these observations suggest that the role of Sox proteins in neural development is highly conserved and, importantly, that SoxN regulates a set of target genes controlled by group B and group C Sox proteins in vertebrates.

### SoxN and Dichaete binding in Sox mutant embryos

In both flies and vertebrates, group B Sox proteins are able to functionally compensate, with single gene mutants showing comparatively weak phenotypes in regions where related proteins are coexpressed. To gain a genomic perspective into this functional redundancy, we generated four additional DamID datasets, assaying the binding of SoxNeuro and Dichaete in wild-type and null mutant embryos lacking the other factor. We refer to these datasets as SoxNDam (SoxN binding in wild type), DDam (Dichaete binding in wild type), D-SoxNDam (SoxN binding in *Dichaete* mutants) and SoxN-DDam (Dichaete binding in *SoxN* mutants). We used null alleles of both *SoxN* and *Dichaete* for the analysis. *SoxN*^*U6-35*^ has a premature stop codon before the DNA binding domain and is a protein null [31,32]. The *Dichaete*^*r72*^ allele has not been molecularly defined but, genetically, it behaves as an amorph in all phenotypic assays [30]. The experiments were performed with hand-picked embryos selected between stages 12 and 17 of embryogenesis to allow sufficient time for the expression of the yellow fluorescent protein (YFP) marker used to identify homozygous mutants. It should be noted that these datasets differ from the SoxN data described above since profiles were generated from non-overlapping stages of development and utilised much smaller sample sizes. While the binding we map in this comparative experiment is not directly comparable with our defined SoxN core dataset or with our previous work defining Dichaete binding, we note that after stage 11 there is still substantial expression of both proteins in the CNS, particularly in the brain and in late segregating trunk neuroblasts, GMCs and their progeny [33,35-37,50]. In addition, post-mitotic cells, such as neurons and glia, expressing the Sox-Dam fusions prior to stage 12 will also be identified in this analysis due to perdurance of the adenine methylation mark.

**Figure 4:**
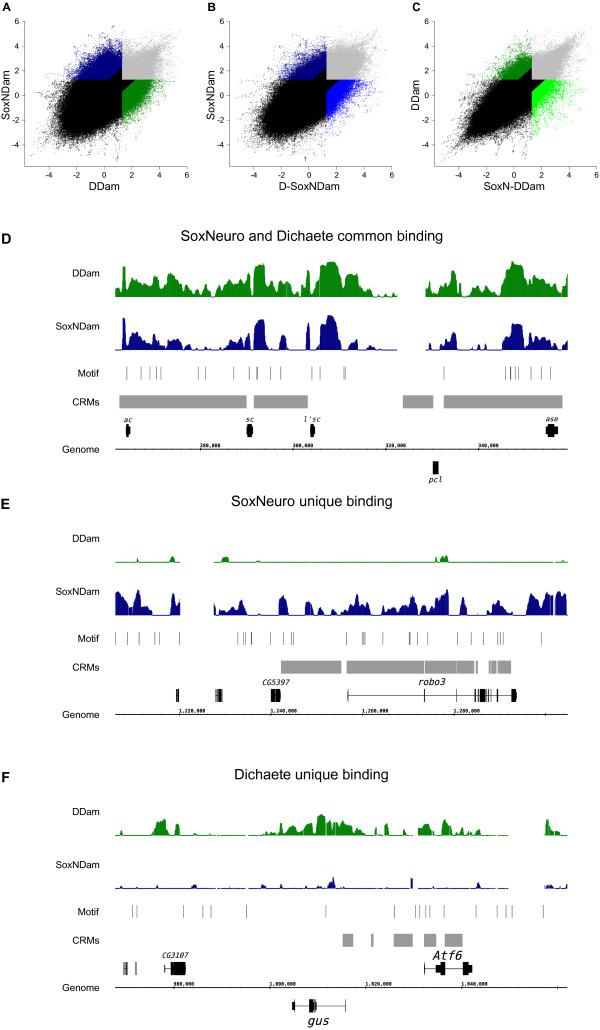
**SoxN and Dichaete differential binding. (A-C)** Differential binding in pairwise comparisons of the SoxNDam, DDam, D-SoxNDam and SoxN-DDam datasets as normalised probe intensities (log_2_ fold change). Light grey areas are probes bound in both datasets, black regions are not bound in either. (A) SoxNDam (dark blue) and DDam (dark green); (B) SoxNDam (dark blue) and D-SoxNDam (light blue); (C) DDam (dark green) and SoxN-DDam (light green). **(D-F)** Representative SoxN and Dichaete binding profiles in wild-type embryos (dark blue and dark green, respectively). Matches to the SoxN binding motif are displayed as thin bars, FlyLight and REDfly enhancers are displayed in light grey. (D) SoxN and Dichaete common binding across the achaete-scute complex. (E) SoxN unique binding in proximity of *robo3*. (F) Dichaete unique binding in the *gus* and *Atf6* region.

Comparing genome-wide profiles by simply overlapping the genomic coordinates of peaks called individually for each dataset is a rather coarse approach and can potentially underestimate binding similarity [67]. In simple pairwise comparisons, peaks with similar height and area may be called in one sample but not the other because of the fixed thresholds applied to each dataset by peak calling algorithms, thus limiting meaningful comparison of binding profiles in different conditions. To overcome this issue, we developed a method to directly compare the normalised ratios of each microarray probe and compute similar and dissimilar genomic regions. We named this tool SimBindProfiles [68] and used it to perform pairwise comparisons between the four datasets and uncover similarly or differentially bound regions (Figure 4A-C). While SimBindProfiles identifies genomic regions that are similar or dissimilar between the profiles being compared, its output is not directly comparable with the binding intervals identified by threshold-based peak calling algorithms. Table 3 summarises the numbers of genomic regions and associated genes obtained with the analysis; all of the corresponding genomic regions, gene sets and corresponding GO:BP enrichments are provided in Additional file 9.

**Table 3:** **SoxN and D binding in wild-type, *D *** **and *SoxN *** **mutant embryos**

**Binding**	**Intervals**	**Genes**
SoxNeuro Dichaete common	2,893	1,890
SoxNeuro unique	3,723	1,649
Dichaete unique	3,506	1,753
SoxNeuro no change	3,720	2,063
SoxNeuro compensatory	794	570
SoxNeuro increased	245	195
SoxNeuro *de novo*	1,893	1,113
SoxNeuro loss	2,497	1,593
Dichaete no change	5,175	2,868
Dichaete compensatory	276	226
Dichaete increased	102	87
Dichaete *de novo*	658	522
Dichaete loss	943	705

Numbers of intervals retrieved by SimBindProfiles and associated genes for the SoxNDam, DDam, D-SoxNDam and SoxN-DDam datasets.

The genome-wide binding profiles of SoxN and Dichaete in wild-type embryos showed extensive overlap (2,893 regions, 1,890 genes), indicating that the proteins often bind at the same locations (Figure 4D). Consistent with their biological roles, the set of common bound genes were enriched for developmental, CNS and transcriptional regulation GO:BP terms (Additional file 10). The set includes major regulators of early CNS specification, including the proneural genes of the achaete-scute complex, the DV patterning TFs encoded by *Dr* and *vnd*, and the NB temporal identity genes (*svp*, *hb*, *kr* and *pdm2*). Altogether, we found that both Sox proteins commonly bound to over a hundred genes encoding TFs with roles in a range of CNS processes. We also identified a large number of genomic regions uniquely bound by either SoxN (Figure 4E; 3,723 regions, 1,649 genes) or Dichaete (Figure 4F; 3,506 regions, 1,753 genes), indicating that their binding pattern is not fully redundant and that they exert at least some of their functions independently of one another. While the gene set uniquely bound by Dichaete was also enriched for GO:BP terms relating to development, CNS functions and transcription, the SoxN unique gene set showed comparatively weak enrichments, although it does contain a set of 95 genes annotated with neuronal differentiation functions (Additional file 10). Thus, Dichaete and SoxN share a common set of targets involved in early and late CNS development. A set of genes with similar functions are uniquely regulated by Dichaete, whereas SoxN unique targets appear to be downstream effectors of basic cellular processes, perhaps indicative of a role in terminal differentiation.

**Figure 5:**
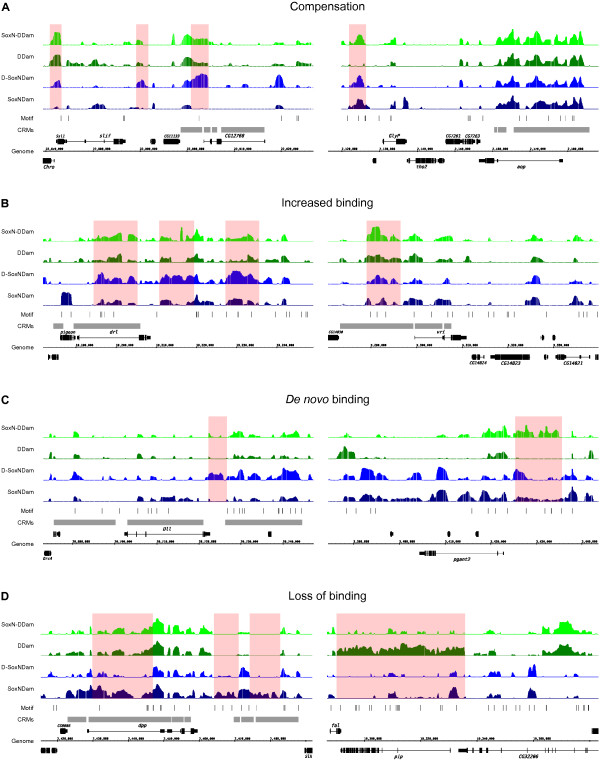
**Profiles of SoxN and Dichaete binding in *Dichaete *and *SoxN *mutant embryos.** Representative SoxN binding profile in *Dichaete* mutant embryos (light blue) and Dichaete binding profile in *SoxN* mutant embryos (light green). Matches to the SoxN binding motif are displayed as thin bars, FlyLight and REDfly enhancers are displayed in light grey. Events of **(A)** compensation, **(B)** increased binding, **(C)***de novo* binding and **(D)** loss of binding are highlighted as red shaded boxes.

To directly address functional redundancy, we examined the binding profiles of SoxN and Dichaete in embryos homozygous for null mutations in the other protein (Figure 4B,C) and identified five different types of event: 1) no change-the binding of each protein was not affected by the loss of the other; 2) compensation - one Sox protein compensated for the loss of the other by binding at locations normally occupied by the latter (Figure 5A); 3) increased binding-in the absence of one Sox protein, the other showed an increase in binding at its normally occupied intervals (Figure 5B); 4) *de novo* binding-in the absence of one Sox protein, the other bound at new regions not normally bound in the wild type (Figure 5C); 5) loss of binding - lack of one Sox protein resulted in loss of binding of the other (Figure 5D).

While at a global level SoxN binding was broadly similar in wild type and *Dichaete* mutants (3,720 regions), a detailed examination identified a variety of changes. In 794 instances SoxN compensated for the loss of Dichaete and increased binding events were identified at 245 genomic locations. SoxN was also often found to bind at new, previously unbound, locations (1,893 instances), but the scenario with the highest impact was loss or strong reduction of binding (2,497 regions), suggesting that Dichaete is often required for the recruitment or retention of SoxN. A considerable proportion (30%, 536 genes) of the 1,753 genes uniquely bound by Dichaete showed evidence of compensation by SoxN at the genic level (considering both compensation and *de novo* but not increased binding events) and these were primarily genes annotated with CNS functions and transcriptional regulation. Interestingly, the majority (58%, 896 genes) of the 1,539 genes that showed a loss of SoxN binding were genes uniquely bound by SoxN. The gene sets for all types of event were associated with moderate to high overrepresentation of GO:BP terms related to CNS development and transcriptional regulation (Figure S6A in Additional file 11).

In contrast, we found that Dichaete binding was less affected by the loss of SoxN. We observed no change in Dichaete binding in SoxN mutant embryos at 5,175 regions, while the other scenarios were observed at much lower frequencies. Dichaete was found to compensate for loss of SoxN at only 276 locations and showed increased binding at 102. *De novo* and loss of binding (658 and 943 instances, respectively) were also similarly reduced. All the associated gene sets were somewhat less enriched for CNS development and gene regulation than their SoxN counterparts (Figure S6B in Additional file 11). Of the 1,649 genes uniquely bound by SoxN, only 14% (232 genes) showed evidence of Dichaete compensation (considering compensation and *de novo* binding events) and these were only weakly enriched for generic GO:BP terms. Finally, we examined the overlap with FlyLight CNS enhancers [64] and found that the number of enhancers bound by both SoxN and Dichaete (621 enhancers, corresponding to 237 genes; Table S6A in Additional file 12) was comparable to the number of enhancers hit by SoxN alone (623 enhancers, 238 genes; Table S6B in Additional file 12) or by Dichaete alone (704 enhancers, 258 genes; Table S6C in Additional file 12), reinforcing the idea that the two factors work independently as well as in concert to direct gene expression in the CNS.

Our analysis of the genome-wide binding comparison of paralogous TFs under mutant conditions strongly supports the hypothesis that group B Sox proteins have both independent and shared functions under normal conditions but can functionally compensate by occupying vacant binding sites when one of the proteins is absent. Importantly, our observations indicate that a considerable fraction of the redundant CNS functions is centred on a core of TFs involved in aspects of neural specification and differentiation, suggesting that both Sox proteins have been maintained in the CNS to provide a degree of robustness to the regulatory networks driving early neurogenesis. Finally, the fact that SoxN targets in *Drosophila* and Sox2 targets in mouse neural cells are well conserved emphasises that SoxB gene functions are essential in the regulatory networks underpinning the most basic aspects of neural development across metazoa.

## Discussion

In this study we performed a genome-wide analysis of the role of the group B Sox gene *SoxN* during *Drosophila* embryonic development and generated a genomic perspective on the functional redundancy of Sox TFs. We identified a high confidence list of SoxN target genes that places SoxN at the heart of the regulatory networks driving neural specification and differentiation. We show an extensive overlap between SoxN and Dichaete genomic binding, but also identify binding indicative of unique functions for each TF during CNS development. In addition, we uncovered unexpected complexity in the relationship between SoxN and Dichaete, with evidence for compensation, dependency and other effects that can potentially explain why the coexpression of group B paralogs has been maintained throughout evolution. The fact that many SoxN targets have orthologs that are targets of Sox2 in mouse NSCs suggests that the roles of group B proteins in the CNS are well conserved. The underlying regulatory networks driving early myogenesis [69], as well as heart [70] and eye development [71], are known to be conserved, and it is likely that more of the core circuitry underpinning basic developmental processes has been maintained throughout animal evolution [72]. Together with the evidence that mammalian group B Sox proteins are able to rescue *SoxN* and *Dichaete* mutant phenotypes [30,31], our data suggest an underlying conservation in the regulatory networks driving early aspects of CNS development across higher metazoans. In addition, the high overlap between SoxNeuro and Sox11 targets suggest SoxNeuro is also involved in late aspects of neural development and differentiation.

As with many other developmentally important TFs, we found that SoxN binds extensively across the genome, and a significant proportion of genes in the genome are affected by its loss. However, many of the genes misregulated in *SoxN* mutants may not be directly controlled by SoxN, but by regulators whose expression is dependent upon SoxN. Consistent with this, we found that many TFs involved in different aspects of neurogenesis and gliogenesis are downregulated in *SoxN* mutants, indicating that a prominent function of SoxN is to promote the expression of genes required for neural development. Some of the genes bound by SoxN may not show significant changes in their expression levels due to functional compensation by Dichaete and thus the network of Sox-related nervous system genes is likely to be even larger. In support of this view, we identified considerable overlap between SoxN and Dichaete binding across the genome, particularly at a number of genes with transcriptional roles in early aspects of neural development, as well as direct evidence of substantial Dichaete compensatory binding in *SoxN* mutants.

Focusing on what we believe to be unambiguous SoxN targets, genes that are both bound by SoxN and change expression in the mutant, we identified a set of genes involved in multiple aspects of embryonic development and morphogenesis. As expected, many of the targets have identified roles in CNS development and form a highly interconnected network, emphasising that SoxN regulates a range of processes, characterized by specific sets of target genes. We can broadly divide SoxN functions into two main categories: early in nervous system development, SoxN controls a battery of genes required for the correct specification of NBs, while at later stages it is involved in regulating the differentiation of both neurons and glia into mature, terminally differentiated cells. The involvement of SoxN in the specific regulation of terminal differentiation is supported by a previously reported analysis [50] that showed both SoxN binding at a set of genes involved in axonal pattering and genetic evidence that SoxN function is directly required for correct axonal pattering.

In particular, early in development *SoxN* promotes the expression of proneural genes *ac* and *ase* while repressing the expression of *hairy*, a known proneural gene repressor [73], thereby driving the acquisition of the neural fate. Of note, SoxN and Dichaete display opposite behaviours during this initial stage of neural specification, since both *ac* and *ase* are partially repressed by Dichaete [32,38]. Dichaete and SoxN interact with the homeodomain proteins Ind and Vnd, which specify neural identity across the DV axis [38,74]. We identified extensive SoxN binding at FlyLight enhancers associated with Vnd, as well as Dr, Egfr and Dichaete, other components of this developmental pathway, and observe changes in Dichaete and Dr expression in *SoxN* mutants. Since Dichaete also displays widespread binding at these DV patterning genes [38,55], it is likely that Dichaete and SoxN act redundantly in this context. The loss of Dr expression in *SoxN* mutants is consistent with this idea, since Dr is restricted to the lateral column of the neuroectoderm where Dichaete is not expressed. In addition, the *de novo* motif discovery search we performed with SoxN binding intervals recovered motifs resembling those reported for Ind/Dr and Vnd [54,57]. We identified over 200 locations in the fly genome containing combinations of Sox and DV patterning TF binding motifs, including regions overlapping 68 FlyLight neural enhancers. In particular, we found co-occurrence of SoxN and Ind/Dr motifs at 43 FlyLight enhancers associated with early neural TF genes (*Dichaete*, *Dr*, s*vp*, *pros* and g*cm*). Together, these data strengthen the view that SoxN, Dichaete and the DV patterning homeodomain TFs interact at regulatory elements in the fly genome to drive establishment of neural fate [33,38]. Since a set of homeodomain proteins also cross-regulate to pattern the vertebrate neural tube and are coexpressed with group B Sox proteins [75], our observations support the view that the DV neural pattering regulatory network has been conserved across evolution [76] and indicate a crucial role for group B Sox proteins in this key aspect of early CNS specification.

Our analysis indicates that the role of SoxN in CNS development extends well beyond early specification events. We identified all the known components of the temporal cascade of TFs regulating neural identity as SoxN targets (*hb*, *Kr*, *nub*, *pdm2*, *cas* and *svp*). We also found highly significant overlaps between SoxN, Dichaete, Hb and Kr binding across the genome, suggesting the possibility of a regulatory feedback network where SoxN promotes the expression of temporal identity factors and then binds with them to orchestrate the differentiation of NBs. Dichaete also shows extensive binding at the genes in the temporal cascade [49] and, consistent with functional redundancy, we found Cas, Hb, Kr, Nub and Pdm2 expression primarily affected in the lateral column of the neuroectoderm in *SoxN* mutants, where Dichaete is not expressed. As we note above, Dichaete has been shown to function in this regulatory cascade [42,77], indicating that group B Sox proteins generally participate in the regulatory networks generating neuronal diversity. We also identified and validated targets implicating SoxN in the regulation of genes controlling self-renewal and asymmetric divisions of NBs and their progeny, ganglion mother cells (*insc*, *numb*, *spdo*, *sna*, *wor* and *esg*[78-84]), and have previously identified roles for Dichaete in these pathways [49]. We note that in vertebrates, B1 proteins are involved in the control of NSC self-renewal and must be downregulated to allow neural differentiation [20-22], further emphasising similarities between fly and vertebrate SoxB functions.

Finally, we identified a substantial number of SoxN targets with known functions in the development and morphogenesis of neuronal axons and dendrites, including *ct*[85], *daw*[86,87], *Dbx*[88], *kn*[85], *lola*[89], *mid*[90], *nerfin-1*[91] and *Sema-1a*[92], thus implicating SoxN in the direct regulation of genes involved in terminal neural differentiation. Our observations support a previous analysis that demonstrated SoxN is expressed in a subset of postmitotic neurons and glia, binds at genes involved in late aspects of neural differentiation and shows axonal phenotypes when mis-expressed or in genetic interactions with its targets (*lola* and *beat1a*) [50]. In addition, in several cases (*daw*, *Dbx*, *lola*, *mid*, *nerfin-1* and *Sema-1a*), mutant phenotypes have been described for SoxN targets that show striking similarities to the lateral axonal phenotypes of *SoxN* mutants [32]. Similarly, we found that SoxN regulates the expression of g*cm* and *gcm2*, the two TFs responsible for the specification and differentiation of all *Drosophila* glial cells [93-95]. We also found that SoxN activates *hkb*, which has been reported to physically interact with Gcm, triggering its autoregulation [96]. Together with the glioblast defects reported in *SoxN* mutants [32], these observations strongly link SoxN to gliogenesis.

Our findings highlight a major difference in the roles group B Sox proteins play in fly and vertebrate CNS development. In vertebrates, the B group has evolved two subclasses, each with specialised and restricted functions: SoxB1 proteins are required for the maintenance of neural precursors, whereas SoxB2 proteins counteract their action, promoting cell cycle exit and neural fate commitment. Differentiation into mature neural cells is promoted by other groups of Sox TFs, primarily groups C (Sox4, Sox11 and Sox12) and E (Sox8, Sox9 and Sox10) [20,97]. In contrast, our data suggest a simpler system in insects, where SoxN and Dichaete are the only Sox genes contributing to the majority of the processes in embryonic neurogenesis, and are reused in different contexts during CNS development. Of the remaining six *Sox* genes in the fly genome, only the group B gene *Sox21a* and the group D gene *Sox102F* show detectable expression in the embryonic CNS, but in both cases expression is relatively late in development and restricted to a handful of specific cells [27]. Thus, in *Drosophila*, all aspects of CNS development, from neural specification through to terminal differentiation, are under the control of group B Sox proteins. The view that SoxB proteins have evolved different roles in insects and vertebrates while maintaining their core functionality is supported by the comparison of gene sets bound by SoxN in *Drosophila* and Sox2 or Sox11 in mouse, which indicates more diversified functions for SoxN in the CNS. Core regulatory genes involved in neural specification and NSC biology are targets of SoxN and, while a set of later target genes involved in neural differentiation are shared by SoxN and Sox11. We also identify a number of intriguing similarities between the roles of Sox proteins in flies and vertebrates during neural development. The contrast between the roles of Dichaete and SoxN in the regulation of proneural genes, with SoxN activating and Dichaete repressing, is reminiscent of the opposing functions shown by vertebrate SoxB1 and SoxB2 subgroups in NSC differentiation [22], and may point to the origin of the group B neofunctionalisation. Similarly, the two SoxB TFs display opposite activity in the regulation of *pros*, with SoxN acting as a transcriptional activator (this study), and Dichaete as a repressor [98]. Aside from these two specific examples, it appears that both SoxN and Dichaete mainly act as partially redundant activators with overlapping roles in early neural specification.

The mechanisms underlying why evolution has maintained substantial overlapping expression of closely related group B Sox proteins has so far remained elusive. While the binding patterns of SoxN and Dichaete in wild-type embryos look broadly similar, supporting a simple model where the two factors act redundantly, a large number of genomic locations display unique SoxN or Dichaete binding. SoxN unique genes appear to be associated with general cellular processes, possibly reflecting a role in terminal differentiation. In the case of Dichaete, we identified a set of uniquely bound TF genes likely to be linked to its roles in segmentation, early midline development and hindgut morphogenesis [34,35]. In line with this, we have also found the expression of many more genes affected in *Dichaete* than in *SoxN* mutant embryos [49]. Since genes showing substantial binding overlap are associated with regulatory networks driving early neural specification, we suggest coexpression has been maintained to provide a degree of robustness to these critical pathways that establish the foundations for early nervous system development. On the other hand, the different binding profiles of *Drosophila* group B paralogs we report here can be interpreted as examples of neofunctionalization.

The analysis of SoxN and Dichaete binding in their respective mutants provided molecular evidence to support the idea that each protein can functionally compensate for the loss of the other. Of interest, we found that SoxN was more able to substitute for Dichaete than vice versa. In some instances, we could explain a lack of compensation by the fact that each of the proteins has unique expression domains; however, since the DamID profiling method we employed to map binding events in the mutants relies on ubiquitous low level expression, lack of coexpression may not be a sufficient explanation. It is possible that regions that do not show compensatory binding reflect SoxN- or Dichaete-specific interactions with cofactors that are not shared between the paralogs, pointing to another level of neofunctionalization. In this respect, we note that SoxN has a role in cuticle patterning that is only partially compensated by Dichaete [99,100], and some of the genes uniquely bound by SoxN have annotated roles in cuticle development. We also have preliminary evidence from rescue experiments that some SoxN neural phenotypes cannot be compensated by Dichaete and that early Dichaete midline functions cannot be fully compensated by SoxN [31].

We uncovered a variety of other binding profile changes indicative of more complex interactions between *Drosophila* group B proteins. We were surprised to find that loss of binding was the most frequently observed event in both mutant conditions, suggesting a high degree of interdependency between the two factors, a novel aspect of *Drosophila* SoxB gene biology. At many locations, Dichaete binding appears to be required for the recruitment or the retention of SoxN, and the opposite situation was also observed, though to a lesser extent. It is possible that these observations indicate obligate heterodimerisation at some sites in the genome as occurs with vertebrate group D and E Sox proteins [101]. Alternatively, it may reflect a requirement for interactions with specific cofactors or for the establishment of a suitable chromatin environment by one Sox protein that is necessary for the binding of the other Sox protein. Given the DNA bending properties of the HMG box DNA binding domain [102], it is possible that some of the loss of binding events we observe in mutant embryos are a reflection of Sox-specific chromatin modifications. We also observed increased and *de novo* binding events in mutant embryos, and in both cases we hypothesise that, in mutant conditions, the remaining Sox protein cannot bind to the vacated locations, but instead occupies nearby open chromatin or increases binding at its normal location to provide sufficient target gene activation.

## Conclusions

Taken together, our studies elucidate the processes coordinated by SoxN during embryogenesis at a genome-wide scale and provide evidence for the conservation of SoxB functions in the core regulatory networks underpinning CNS development. We show that, unlike mammalian SoxB1 proteins, SoxN activity is involved in all aspects of neural development, from the initial specification of NBs to their terminal differentiation into mature neural cells. This suggests that *Drosophila* group B proteins may represent baseline metazoan Sox functions that have been elaborated and diversified as the family expanded in vertebrates. Finally, we provide a detailed genomic perspective on functional redundancy between coexpressed paralogous TFs. We describe genomic regions associated with both redundant and independent functions, uncover evidence for extensive interdependency between the two paralogs and identify key regulatory genes subject to functional compensation, suggesting that redundancy supports the robustness of developmental gene regulatory networks.

## Materials and methods

### Fly husbandry and embryo collection

Fly stocks were obtained from the Cambridge Genetics Department Stock Collection or from the Bloomington Stock Center. Oregon-R was used as wild type. Fly stocks were maintained at 18°C or 25°C on standard cornmeal medium and dried yeast. Embryo collections were performed at 25°C in collection cages on grape agar juice plates supplemented with fresh yeast paste. For all experiments, embryos were collected in Nytex baskets, dechorionated for 5 minutes in 50% bleach and washed thoroughly with water.

### Gene expression experiments

Embryos from *SoxN*^*U6-35*^/*CyO, twi*-*Gal4 UAS*-*EGFP* X *Df(2 L)ED647*/*CyO, twi*-*Gal4 UAS*-*EGFP* crosses were used to generate gene expression profiles. For stage 10 and older, approximately 200 *SoxN*^-/-^ and *SoxN*^*+/-*^ embryos per replicate were selected under a fluorescence dissecting microscope on the basis of green fluorescent protein (GFP) expression. For earlier stages of development, a PCR-based method for genotyping single embryos was employed with 12 mutant and control embryos used for each replicate [103]. Microarray hybridization using four biological replicates was performed using our standard protocols [104], with full details provided in the Additional file 13 materials and methods. Scanned images were imported into Dapple [105] for spot finding and quantification, raw data were normalised with the variance stabilization method [106] and statistical analysis of differential expression was carried out using the limma Bioconductor package [107].

### Genome-wide binding assays

Generation of the *SoxNDam* transgenic line is described in the Additional file 13 materials and methods, and the *DichaeteDam* line was previously described [49]. Embryos from *Dam*, *SoxNDam*, *DDam*, *SoxN*^*U6-35*^/*CyO, Dfd*-*YFP*; *DDam* and *SoxNDam*; *D*^*r72*^/*TM6B, Dfd-YFP* stocks were collected and processed for hybridisation to Nimblegen tiling arrays (GEO platform 15641) using minor modifications to the protocol of Vogel and colleagues [108]. For mapping in the wild type we used approximately 2.5 mg dry weight of embryos per replicate; in the case of binding in mutants, approximately 200 YFP-negative embryos at the appropriate stages were selected under a fluorescence dissecting microscope. ChIP followed by hybridisation to Nimblegen tiling arrays was performed with minor modifications to the method described by Sandmann and colleagues [109], as detailed in the Additional file 13 materials and methods. Three biological replicates were performed for all DamID and ChIP experiments. Tiling arrays were quantified using Nimblescan and quantile normalisation was applied to the raw data before using the Ringo Bioconductor package [110] for peak calling at different FDRs. Window score (SGR) and binding interval (BED) files were visualised with the Integrated Genome Browser [111]. The comparative analysis of SoxN and Dichaete binding in wild-type and mutant embryos was performed after all datasets were quantile normalised together. The resulting intensity ratios were used to perform pairwise and three-way comparisons between the datasets with SimBindProfiles [68] as detailed in the Additional file 13 materials and methods.

### Other analysis

The BEDTools suite [112] was used for operations with BED files. Assignment of intervals to genes was performed using a custom script identifying the closest TSS in a 10 kb window. If no TSSs were found, the interval was assigned to the closest gene boundary in the same 10 kb window or left otherwise unassigned. GO:BP term enrichment analyses were performed using the BiNGO Cytoscape plugin [113] and corrected for multiple hypothesis testing with the Benjamini-Hochberg method. The HOMER software suite [114] was utilised for both *de novo* motif discovery and to find enrichment of previously known motifs. Mapping *de novo* motif matches to the *Drosophila* genome was done using FIMO at a *P*-value cutoff of 1E-4 [56]. Embryonic binding datasets from the BDTNP [58,59] and modENCODE [60,61] projects were used to identify TF or chromatin feature overlaps using a subsampling-based approach [60,115]. FlyExpress [65] was used for the production of genome-wide expression maps. For network analysis, the whole DroID database [66], with the exception of TF-gene, microRNA-gene and predicted protein-protein interactions was used. The resulted network was imported into Cytoscape [116] and used for further analysis.

### Immunohistochemistry and *in situ* hybridisation

Embryos from *SoxN*^*U6-35*^/*CyO, twi-Gal4 UAS-EGFP* X *Df(2 L)ED647*/*CyO* and *twi-Gal4 UAS-EGFP* or *Kr-Gal4*/*CyO* X *UAS-SoxN* were collected and processed for antibody staining essentially as described by Patel *et al*. [117] or for *in situ* hybridisation as described by Tautz and Pfeifle [118]. Full details, including the primary antibodies used and their dilutions, are provided in the Additional file 13 materials and methods.

### Data access

All gene expression and ChIP microarray data described in this paper are available from NCBI Gene Expression Omnibus (GEO) in the Superseries accession [GEO:GSE47338].

## Abbreviations

BDTNP: Berkeley Drosophila Transcription Network Project; BP: biological process; ChIP: chromatin immunoprecipitation; CNS: central nervous system; CRM: *cis*-regulatory module; DamID: DNA adenine methyltransferase identification; DV: dorsoventral; FDR: false discovery rate; GFP: green fluorescent protein; GO: Gene Ontology; modENCODE: Model Organism Encyclopedia of DNA Elements; NB: neuroblast; NSC: neural stem cell; TF: transcription factor; TSS: transcription start site; UTR: untranslated region; YFP: yellow fluorescent protein.

## Competing interests

The authors declare that they have no competing interests.

## Authors’ contributions

EF and SR conceived and designed the experiments; EF performed the experiments; EF, BF and SR analysed the data; EF, BF and SR contributed reagents/material/analysis tools. EF and SR wrote the paper. EF, BF and SR read and approved the final manuscript.

## Supplementary Material

Additional file 1: Table S1 Expression data, gene lists and Gene Ontology enrichments for genes differentially expressed in *SoxN* mutants. Click here for file

Additional file 2: Figure S1 Comparison of the different SoxN gene expression and binding datasets. Click here for file

Additional file 3: Table S2 Genomic coordinates, gene lists and GO:BP enrichments of the SoxN core dataset. Click here for file

Additional file 4: Figure S2 General features of SoxN binding. Click here for file

Additional file 5: Figure S3 Comparison of SoxN genome-wide binding with that of other TFs, chromatin-binding proteins and histone modifications. Click here for file

Additional file 6: Table S3 Genomic coordinates, gene lists, GO:BP enrichments for SoxN direct targets and tables of upregulated, downregulated and variably expressed direct targets. Click here for file

Additional file 7: Figure S4 Expression of SoxN direct targets in wild-type and *SoxN* mutant embryos. Click here for file

Additional file 8: Table S4 Gene lists and GO:BP enrichments for genes bound by SoxN and Sox2 or Sox11. Click here for file

Additional file 9: Table S5 Genomic coordinates, gene lists and GO:BP enrichments of SoxN and Dichaete binding in wild type and *Dichaete* and *SoxN* mutants. Click here for file

Additional file 10: Figure S5 Differential enrichment of genes targeted by SoxN and Dichaete. Click here for file

Additional file 11: Figure S6 Differential enrichment of genes associated with the five types of events observed in *SoxN* and *Dichaete* mutants. Click here for file

Additional file 12: Table S6 SoxN and Dichaete binding intervals targeting FlyLight enhancers with reported CNS expression. Click here for file

Additional file 13 Supplementary Methods and Legends. Click here for file
