# Supplementary figures and images for: SoxNeuro orchestrates central nervous system specification and differentiation in Drosophila and is only partially redundant with Dichaete
Source: Genome Biol. 2014 May 30;15(5):R74. doi: 10.1186/gb-2014-15-5-r74 (PMC4072944; doi:10.1186/gb-2014-15-5-r74)

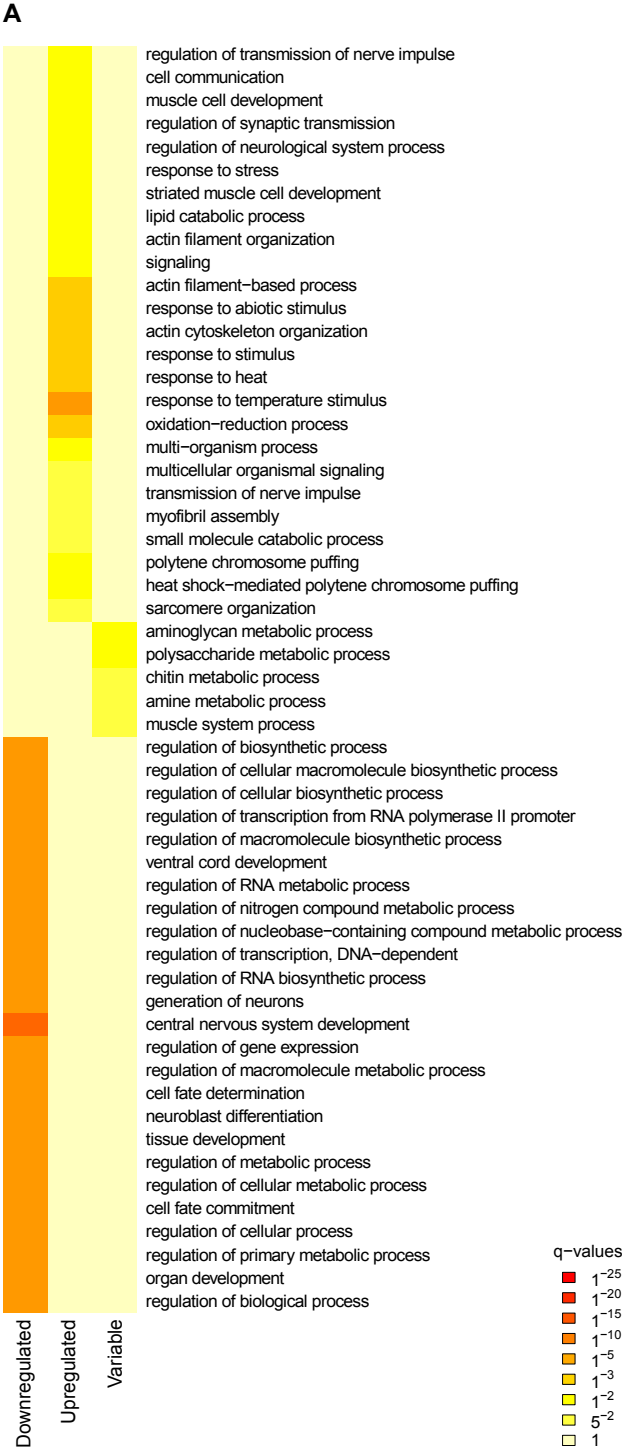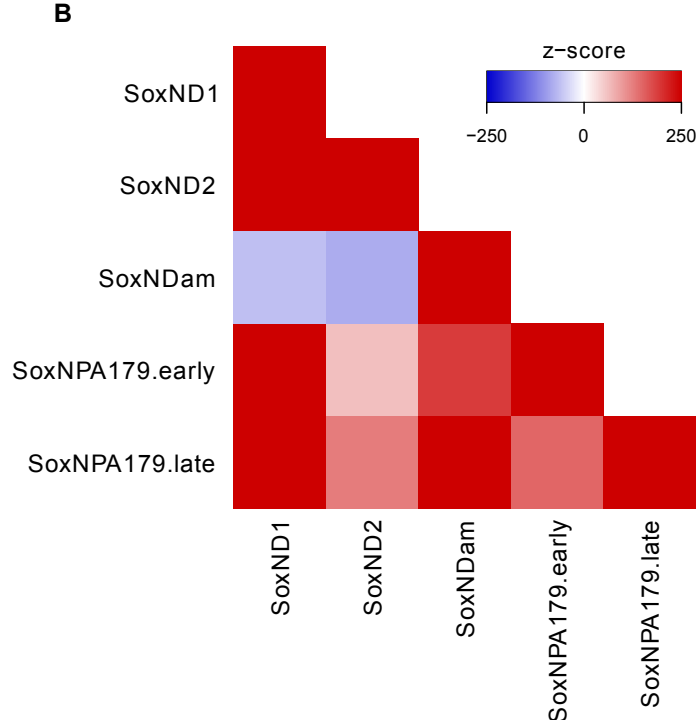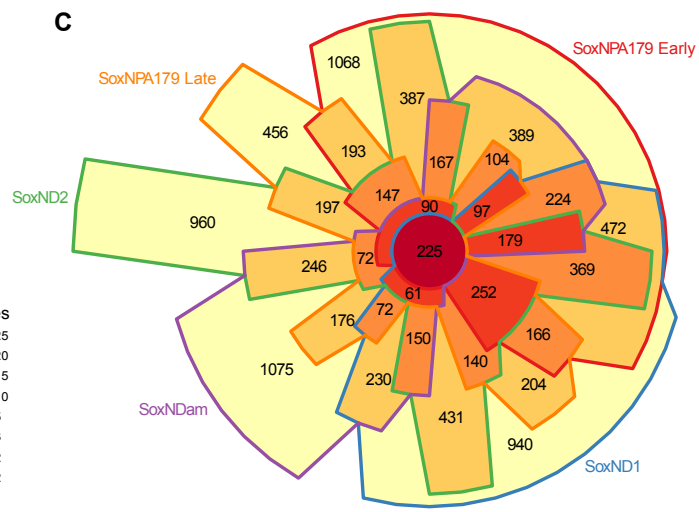

Supplement: Additional file 2: Figure S1 — Comparison of the different SoxN gene expression and binding datasets. [file gb-2014-15-5-r74-S2.pdf]

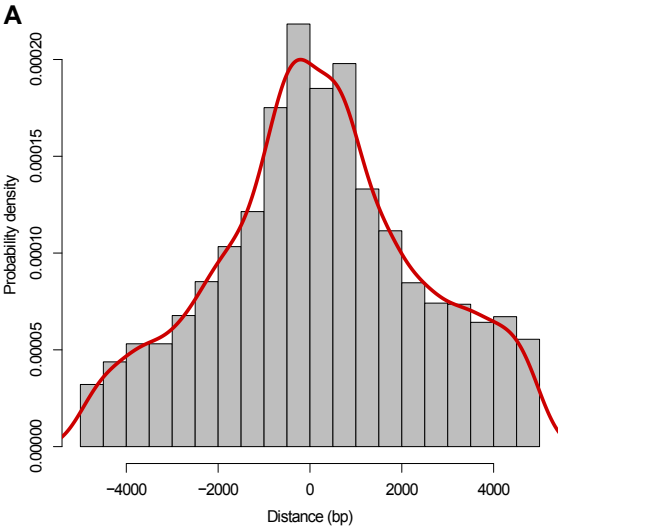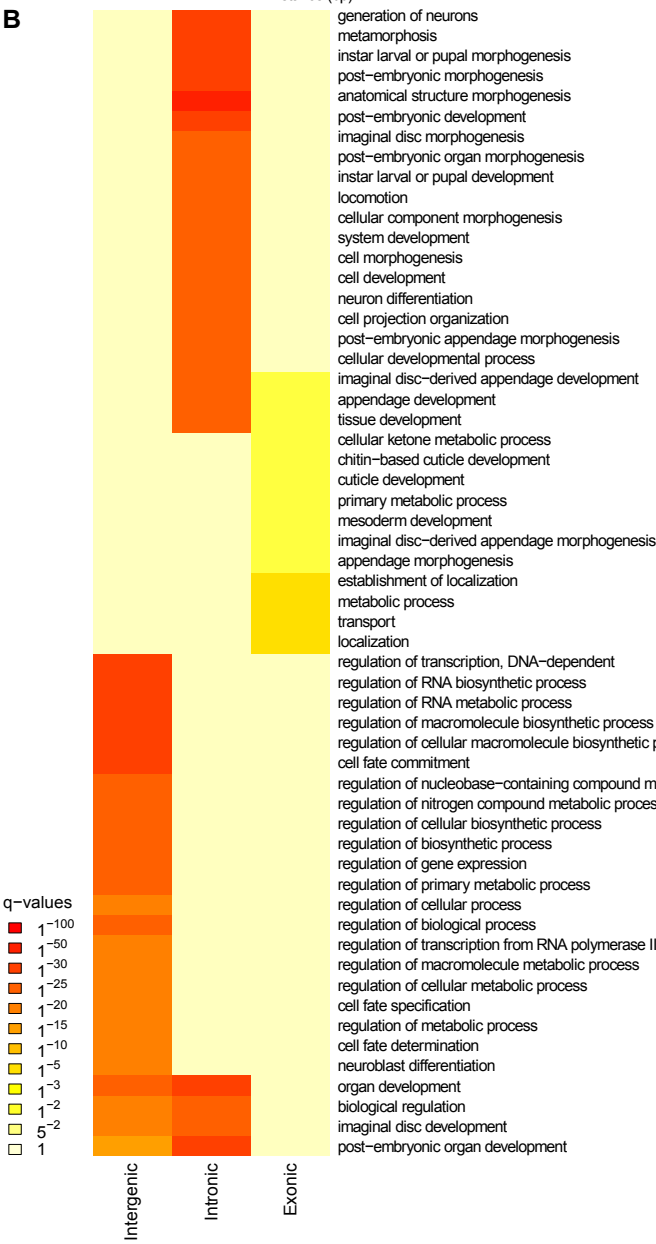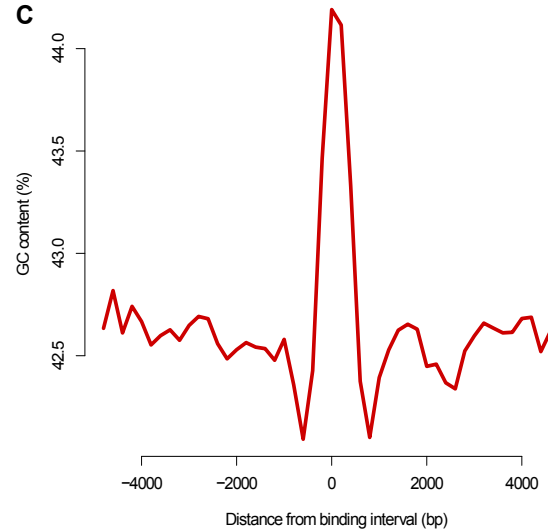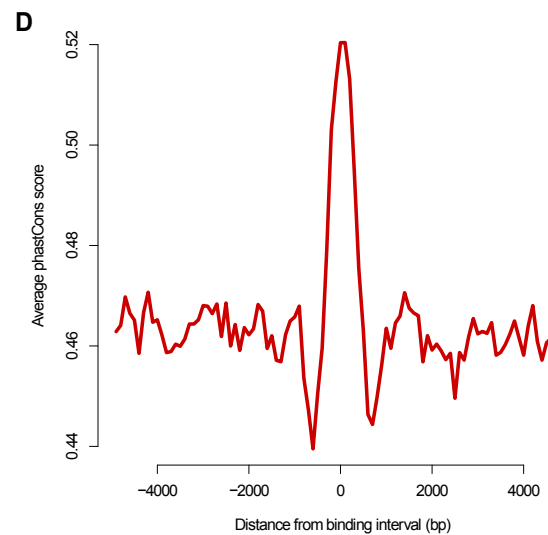

**E**

| Motif | TF    | p-value |
|-------|-------|---------|
|       | mSox2 | 1e-27   |
|       | mSox3 | 1e-25   |
|       | mSox6 | 1e-23   |

Supplement: Additional file 4: Figure S2 — General features of SoxN binding. [file gb-2014-15-5-r74-S4.pdf]

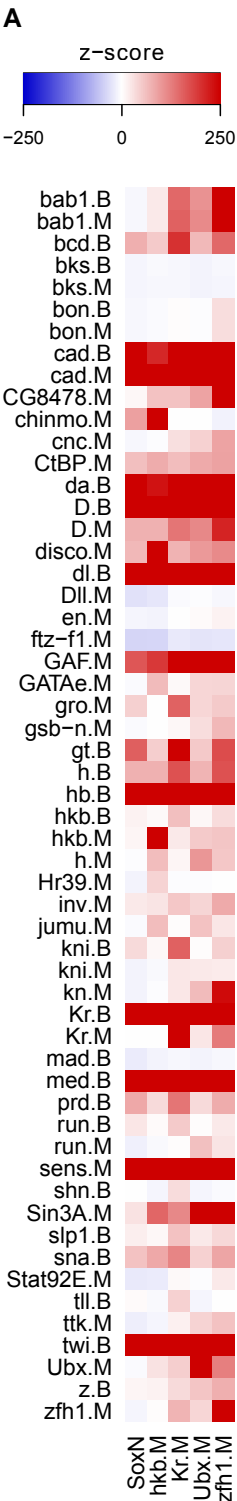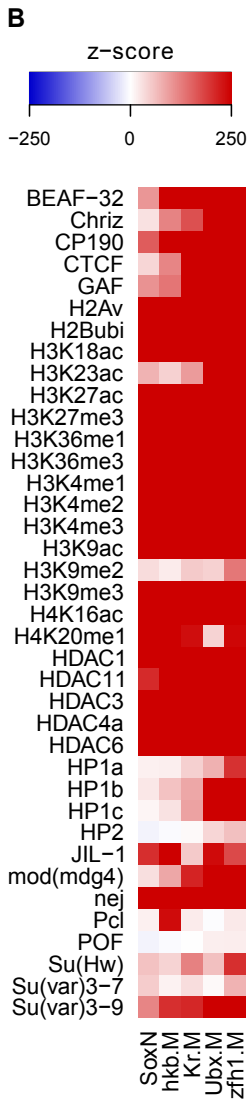

Supplement: Additional file 5: Figure S3 — Comparison of SoxN genome-wide binding with that of other TFs, chromatin-binding proteins and histone modifications. [file gb-2014-15-5-r74-S5.pdf]

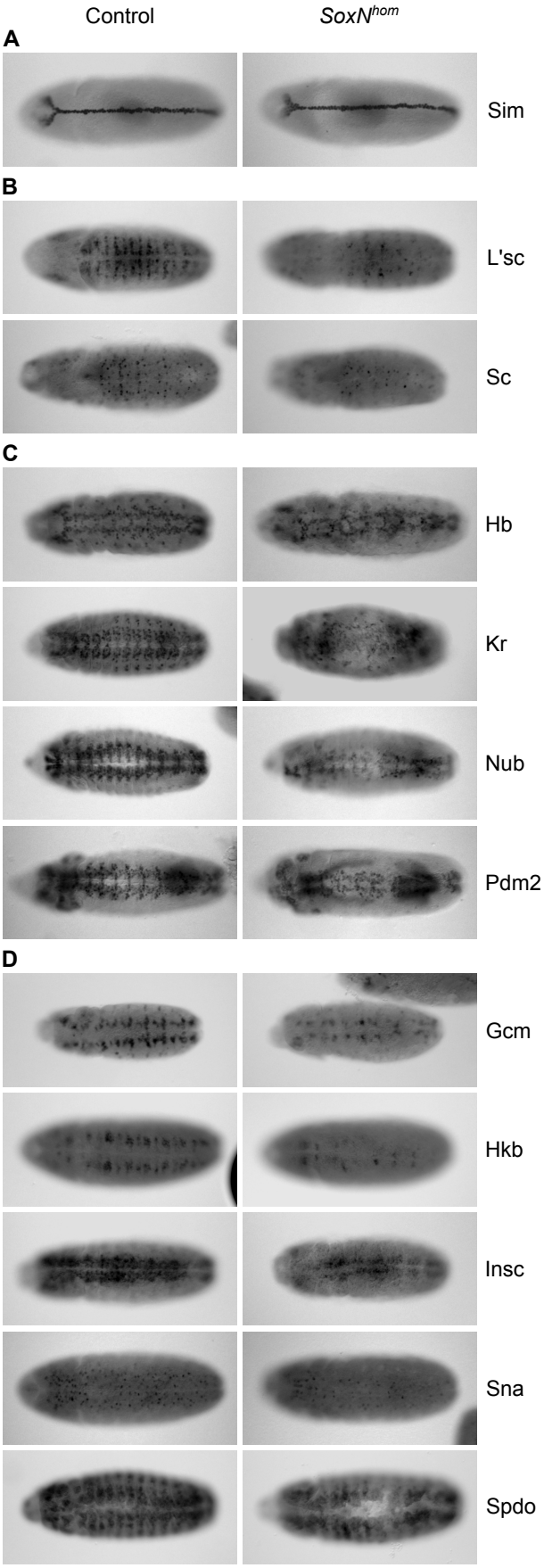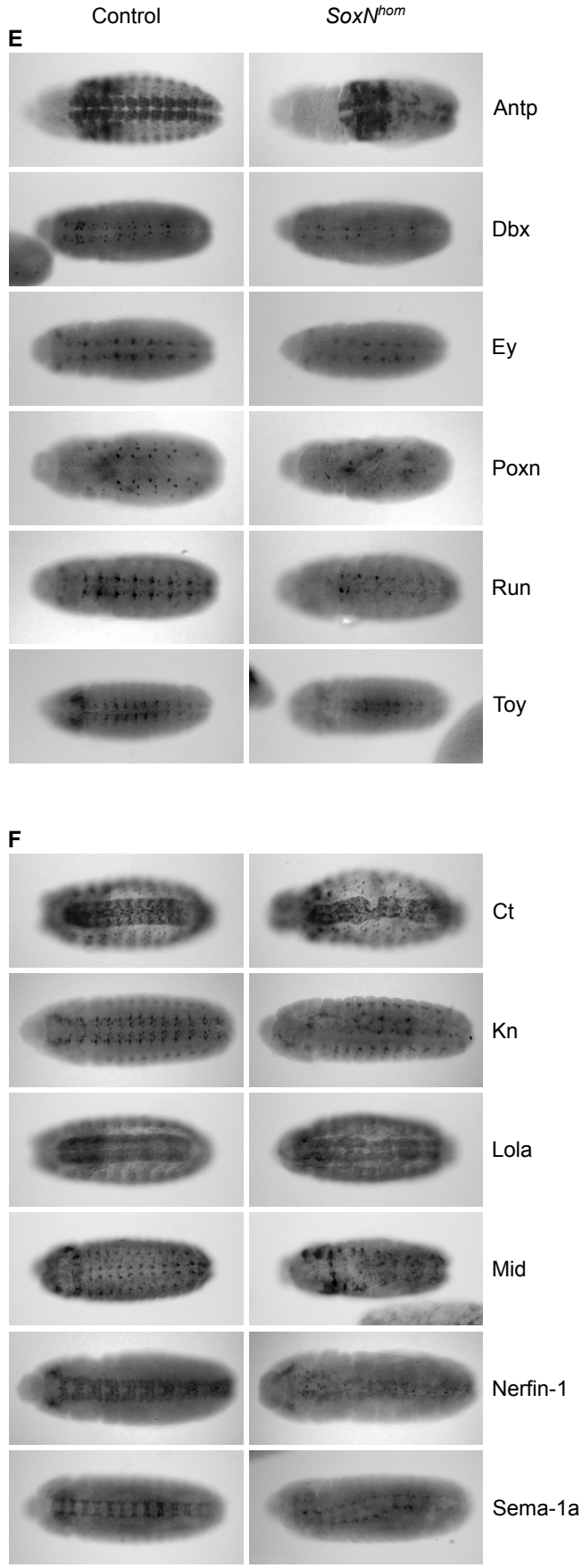

Supplement: Additional file 7: Figure S4 — Expression of SoxN direct targets in wild-type and SoxN mutant embryos. [file gb-2014-15-5-r74-S7.pdf]

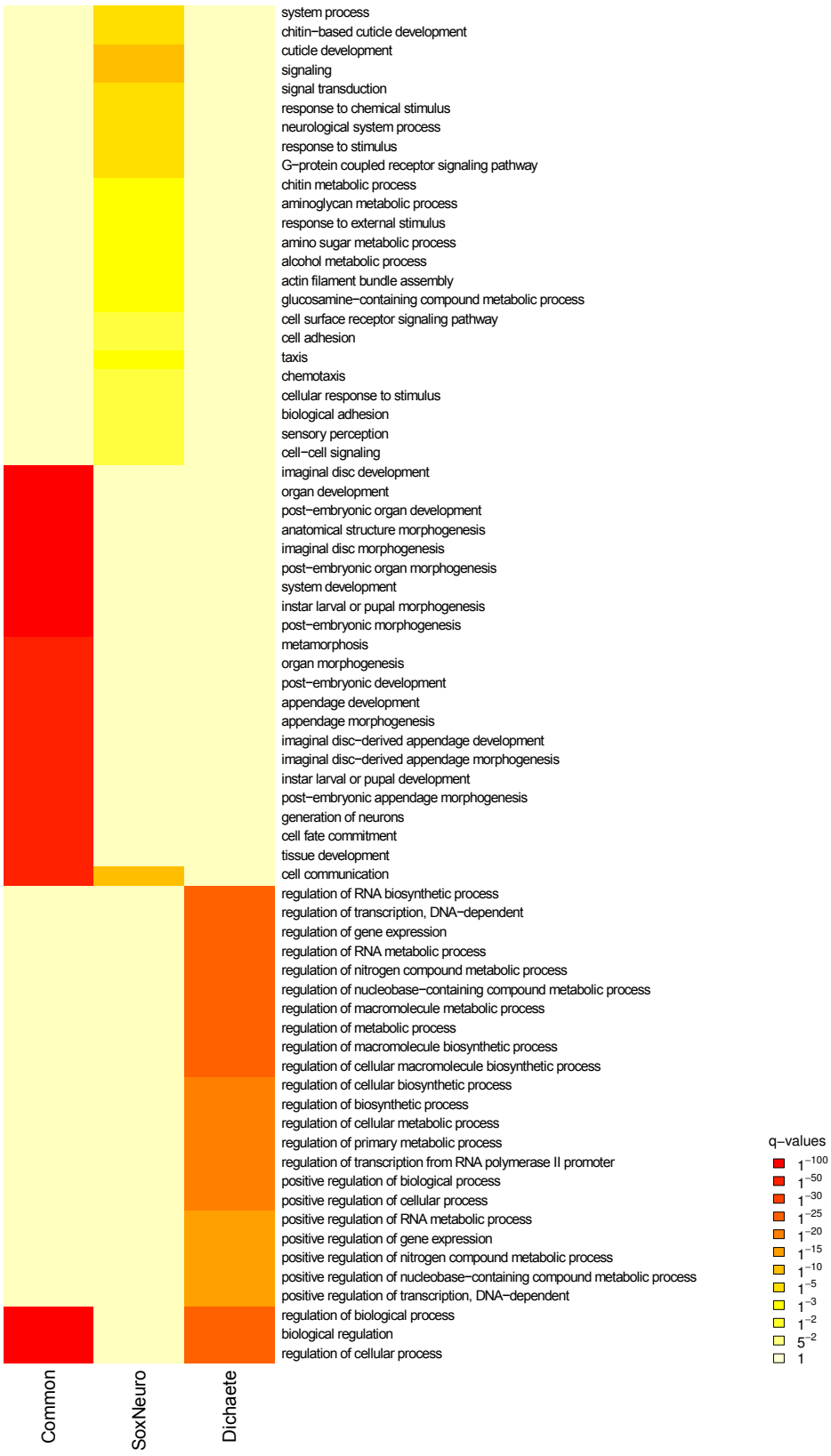

Supplement: Additional file 10: Figure S5 — Differential enrichment of genes targeted by SoxN and Dichaete. [file gb-2014-15-5-r74-S10.pdf]

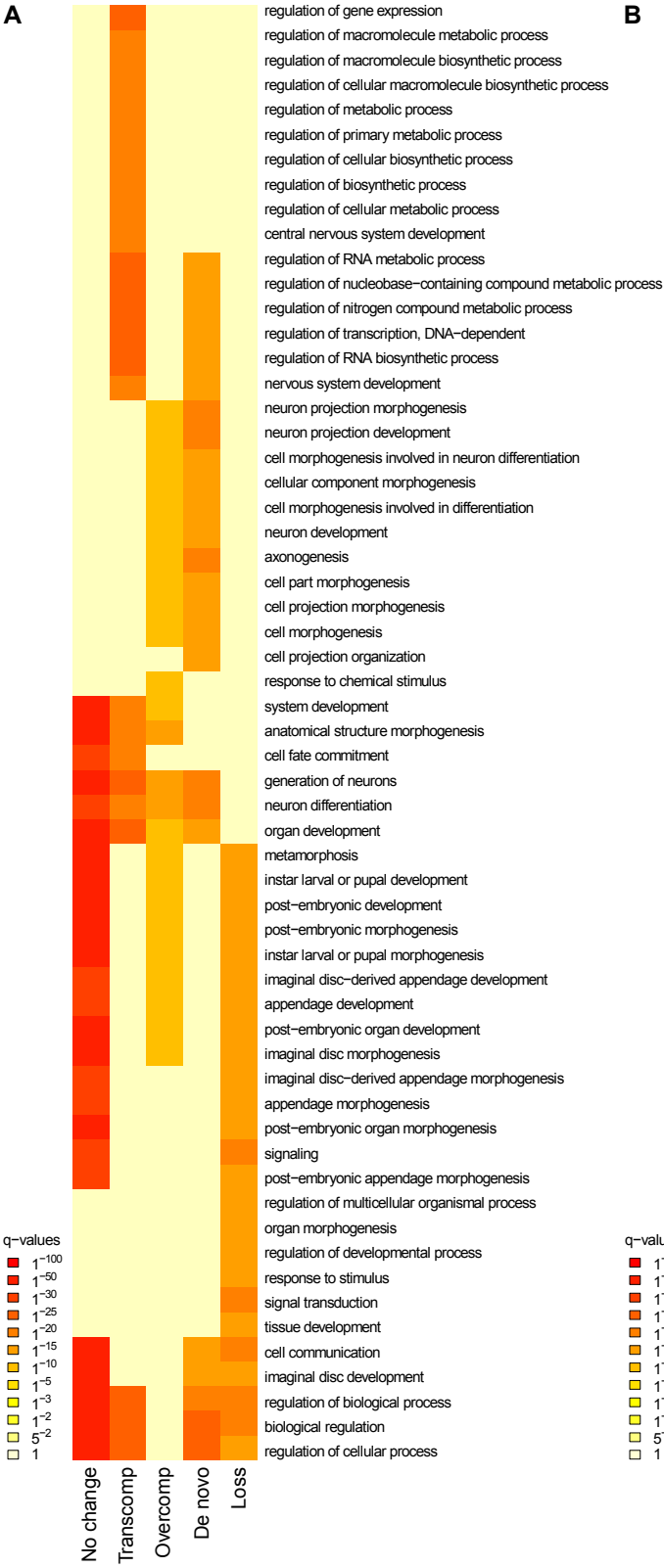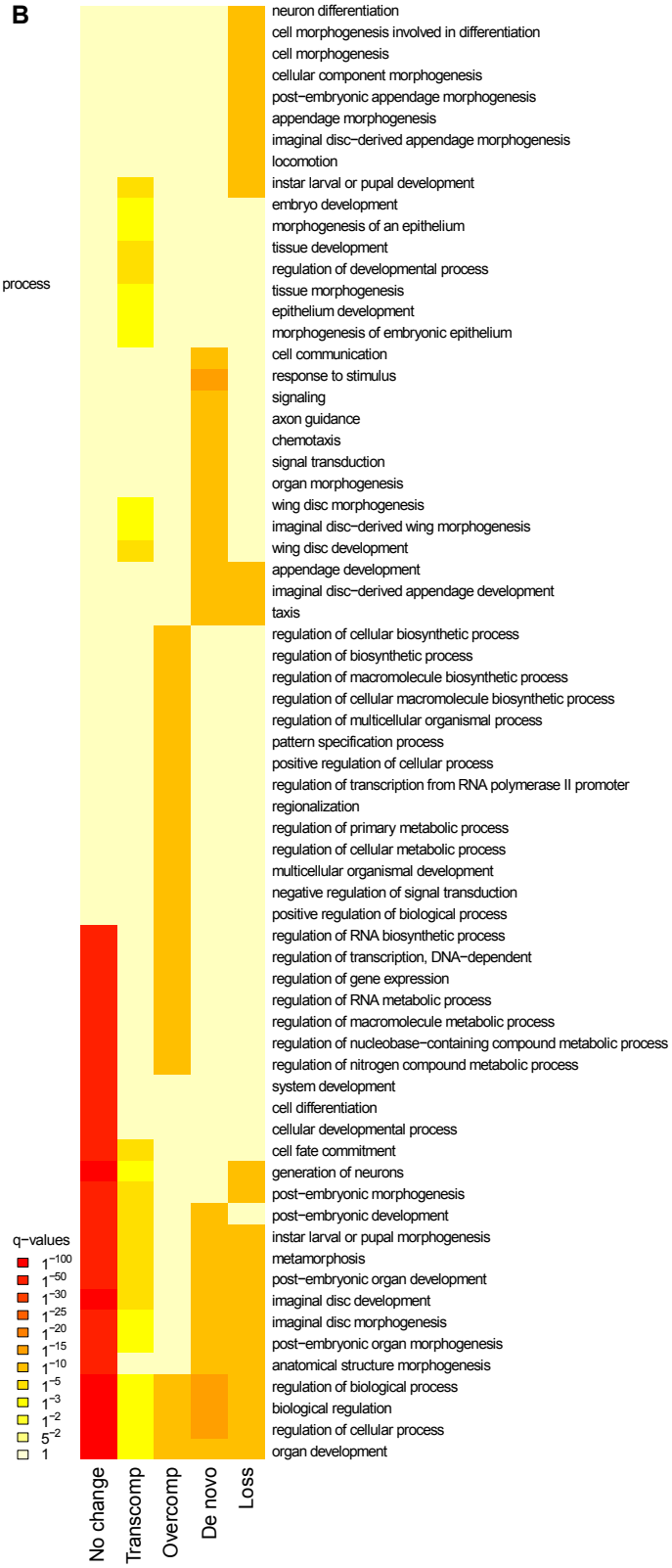

Supplement: Additional file 11: Figure S6 — Differential enrichment of genes associated with the five types of events observed in SoxN and Dichaete mutants. [file gb-2014-15-5-r74-S11.pdf]
