# Supplementary material for: SoxNeuro orchestrates central nervous system specification and differentiation in Drosophila and is only partially redundant with Dichaete
Source: Genome Biol. 2014 May 30;15(5):R74. doi: 10.1186/gb-2014-15-5-r74 (PMC4072944; doi:10.1186/gb-2014-15-5-r74)
Supplement: Additional file 13 — Supplementary Methods and Legends. [file gb-2014-15-5-r74-S13.pdf]

SoxNeuro orchestrates central nervous system specification and differentiation in *Drosophila* and is only partially redundant with *Dichaete*.

Enrico Ferrero, Bettina Fischer and Steven Russell

Department of Genetics, University of Cambridge, Downing Street, CB2 3EH, Cambridge, United Kingdom and Cambridge Systems Biology Centre, University of Cambridge, Tennis Court Road, CB2 1QR, Cambridge, United Kingdom.

EF: ef300@cam.ac.uk;

BF: bef22@cam.ac.uk;

SR: sr120@cam.ac.uk, corresponding author.

ADDITIONAL MATERIALS AND METHODS

Generation of the SoxNDam transgenic line

SoxN was first PCR-amplified with specific primers from the LD44245 clone (Drosophila Genomics Resource Center) and then cloned (EcoRI-NotI) into pCMycDam. SoxN-Myc-Dam was then excised and cloned (EcoRI-XbaI) into pUAST. Plasmid DNA was injected into w^{1118} embryos together with a helper plasmid carrying P element transposase [1].

Gene expression

Embryos from *SoxN$^{U6\text{-}35}$/CyO, twi-Gal4 UAS-EGFP* X *Df(2L)ED647/CyO, twi-Gal4 UAS-EGFP* crosses were collected, dechorionated and kept in PBS on ice. For stage 10 and older, approximately 200 *SoxN$^{-/-}$* and *SoxN$^{+/-}$* embryos per replicate were selected under a fluorescence dissecting microscope on the basis of GFP expression. The standard FlyChip protocol was used for the subsequent steps [http://www.flychip.org.uk]. RNA was recovered using TRIzol extraction followed by ethanol precipitation. RNA was then retrotranscribed into cDNA and a second strand synthesis reaction was performed to yield double-stranded cDNA. Amplification and labelling of cDNA with Cy3 and Cy5 was performed using the Klenow fragment of DNA polymerase and random priming. For earlier stages of development, a PCR-based method for genotyping single embryos was employed [2]: Individual embryos were squashed with a tip in 14.5 µl of PBS, and 3.5 µl were used for genotyping with a PCR assay designed to amplify part of EGFP and a region of chromosome *X*. The remaining 11 µl were stored at -80C after the addition of 30 µl of TRIzol (Life Technologies), and then combined together or discarded after the genotype of each sample was known. For each replicate, 12 homozygous mutant and 12 control embryos were amplified using the SMART method (Clontech) prior to labelling for microarray analysis. In all cases, sample and control DNAs were precipitated, combined together and denatured prior to loading onto FlyChip FL003 long oligonucleotides arrays

(GEO platform GPL14121). Slides were hybridized for 16 hours at 51°C, washed and scanned using a GenePix 4000B scanner. Each experiment was performed in quadruplicate.

DamID

Embryos from Dam, *SoxNDam, DDam, SoxN$^{U6\text{-}35}$/CyO, Dfd-YFP; DDam* and *SoxNDam; D^{r72}/TM6B, Dfd-YFP* stocks were collected, dechorionated and kept in PBS on ice. Approximately 2.5 mg dry weight of embryos per replicate were used for the SoxNDam reference profile. For the comparative experiments examining SoxN and Dichaete binding in mutants, approximately 200 embryos per replicate were collected from the *SoxN$^{U6\text{-}35}$/CyO*, *Dfd-YFP; Ddam: SoxNDam; D^{r72}/TM6B, Dfd-YFP: Ddam: SoxNDam* and *Dam* lines. For the mutants, YFP negative embryos were selected under a fluorescence dissecting microscope. Samples were then processed according to the method of Vogel and colleagues [3]. Genomic DNA was extracted using the Qiagen DNeasy blood and tissue kit and precipitated with ethanol. DNA was digested overnight at 37°C with DpnI to cut methylated GATC sequences and the fragments were ligated to a double-stranded adapter oligonucleotide. Ligation products were subjected to DpnII digestion to remove unmethylated GATC sequences and amplified by PCR. Klenow labelling by random priming was used to incorporate Cy3 and Cy5 into the DNA. After combining samples with Dam-only controls, DNA was loaded onto NimbleGen Drosophila melanogaster Whole Genome 2.1M tiling arrays (GEO platform 15641). For each experiment, three arrays, corresponding to three biological replicates, were hybridized overnight at 42°C, then washed and scanned the following day.

ChIP-on-chip

ChIP followed by microarray hybridization was performed as described by Sandmann and

colleagues [4]. SoxND1 and SoxND2 are polyclonal antisera raised in rabbit immunized with protein fragments corresponding to amino acids 2-92 and 317-417 of SoxN, respectively, and were produced by the modENCODE consortium (a gift of N Negre and K. White). SoxNPA179 is an affinity-purified rabbit polyclonal antibody designed against a 506-LHYQTDSPDLQQQHQS-521 peptide at the C-terminal of SoxN, commissioned from Eurogentec. A mouse monoclonal antibody against βGal (40-1a, Developmental Studies Hybridoma Bank, DSHB) was used for control immunoprecipitations. Following dechorionation, approximately 2.5 mg wet weight of embryos per replicate were crosslinked with formaldehyde and lysed to extract protein-DNA complexes. After sonication, chromatin average size (~500 - 1000 bp) was checked by electrophoresis. Chromatin was then incubated overnight with protein A agarose beads, salmon sperm DNA and anti-βGal (control) or anti-SoxN antibodies. After extensive washes, crosslinking was reversed by incubation at 65°C for 6 hours and DNA isolated through phenol-chloroform extraction. DNA was amplified with two rounds of ligation-mediated PCR, then labelled by Klenow amplification and random priming, and hybridized onto NimbleGen Drosophila melanogaster Whole Genome 2.1M tiling arrays (GEO platform 15641) overnight at 42°C. Each experiment was performed and hybridized in triplicate.

Data analysis

Gene expression arrays were processed according to established FlyChip pipelines [http://www.flychip.org.uk]. Scanned images were imported into Dapple [5] for spot finding and quantification, raw data was normalised with the variance stabilization method (VSN) [6]. Experiments performed at different stages of embryonic development were analysed together with the limma Bioconductor package [7] to retrieve probes differentially expressed ($p \leq 0.05$) over the timecourse. Nimblescan was used to quantify features on the scanned images of DamID and ChIP microarrays. Quantile normalisation was applied

to the raw data before using the Ringo Bioconductor package [8] for peak calling at different FDRs. Window scores (SGR) and binding intervals (BED) files were visualised with the Integrated Genome Browser, [9]. The SoxNDam, DDam, SoxN-DDam and D-SoxNDam DamID experiments were quantile normalised together and the resulting ratios were used to perform pairwise and three-way comparisons between the datasets with SimBindProfiles [10]. This tool does not rely on peak calling, but directly compares binding profiles, allowing the retrieval of commonly and differentially bound regions between datasets, as well as trans- and over-compensation events. The BEDTools suite [11] was used for operations with BED files. Assignment of intervals to genes was performed using a custom script identifying the closest TSS in a 10 kb window. If no TSSs were found, the interval was assigned to the closest gene boundary in the same 10 kb window or left otherwise unassigned. GO:BP terms enrichment analyses were performed using BiNGO [12], a Cytoscape plugin. Terms were considered significant if their p-value, corrected for multiple hypothesis testing with the Benjamini-Hochberg method, was below a 0.05 threshold. The HOMER software suite [13] was utilised for both *de novo* motif discovery and to find enrichment of previously known motifs. Mapping *de novo* motif matches to the *Drosophila* genome was done using FIMO at a p-value cut off of 1E-4 [14]. To assess the similarity of binding datasets, an algorithm performing pairwise comparisons of BED files and relying on a subsampling-based approach was employed [15, 16]: Embryonic binding datasets from the BDTNP (Berkeley Drosophila Transcription Network Project) [17] and modENCODE (Model Organism Encyclopedia of DNA Elements) [16, 18] projects were used. FlyExpress [19] was used for the production of genome-wide expression maps. For network analysis, the whole DroID database [20], with the exception of TF-gene, microRNA-gene and predicted protein-protein interactions was used. The resulted network was imported into Cytoscape [21] and used for further analysis.

SimBindProfiles

The SimBindProfiles Bioconductor package [10] identifies common and unique binding regions in genome tiling array data. This package does not rely on peak calling, but directly compares binding profiles processed on the same array platform. It implements a simple threshold approach, thus allowing retrieval of commonly and differentially bound regions between datasets as well as events of trans- and over-compensation. In order to identify probes or regions that are similarly or differentially bound between the data sets, we implemented the twoGaussiansNull method established in the Ringo package to set a bound cut-off, probes above this threshold are considered "bound". When comparing two datasets, a probe is considered uniquely bound in one data set if it is bound above a diff.cutoff threshold (in our case we used a diff.cutoff threshold of 75% of the bound.cutoff of the other data set). The R package is available from BioConductor [http://www.bioconductor.org/packages/2.14/bioc/html/SimBindProfiles.html].

Immunohistochemistry

After collection and dechorionation, antibody staining of embryos from *SoxN*$^{U6\text{-}35}$*/CyO, twi-Gal4 UAS-EGFP* X *Df(2L)ED647/CyO, twi-Gal4 UAS-EGFP* or *Kr-Gal4/CyO* X *UAS-SoxN* crosses was carried out essentially as described by Patel [22]. The following primary antibodies were added at the indicated concentration and left rolling overnight at 4°C: anti-Ac (1:3, [23]), anti-Antp (1:20, [24]), anti-Ase (1:400, [25]), anti-Cas (1:500, [26]), anti-Ct (1:100, [27]), anti-D (1.100, [28]), anti-Dbx (1:500; [29]), anti-Hb (1:200, [30]), anti-Hkb (1:250, [31]), anti-Insc (1:500, [32]), anti-Kn (1:500, [33]), anti-Kr (1:500, [34]), anti-L'sc (1:300, [35]), anti-Lola (1:50, [36]), anti-Mid (1:500, [37]), anti-Nerfin-1 (1:1000, [38]), anti-Nub (1:250, [39]), anti-Pdm2 (1:5, [40]), anti-Poxn (1:100, [41]), anti-Pros (1:4, [42]), anti-Run (1:200, [34]), anti-Sc (1:300, [35]), anti-Sema-1a (1:1000, [43]), anti-Sna (1:100, [31]), anti-Spdo (1:1000, [44]) and anti-Wor (1:1000, [32]). Following extensive washes,

biotinylated secondary antibodies (Vector Labs) were added at a 1:200 concentration and left for 2 hours rolling at room temperature. After washes, detection was performed by incubating embryos for 1 hour with the Vectastain ABC system. Colour development was achieved by addition of DAB and H2O2 to embryos in watchglasses. After washing, embryos were left to sink in 50% and then 70% glycerol, mounted on slides and observed with a Zeiss Axioplan microscope.

RNA in situ hybridization

RNA in situ hybridization to embryos from *SoxN$^{U6\text{-}35}$/CyO, twi-Gal4 UAS-EGFP* X *Df(2L)ED647/CyO, twi-Gal4 UAS-EGFP* crosses was performed as described by Tautz and Pfeifle [45]. cDNA clones for *ey*, *gcm* and *toy* were obtained from the Drosophila Gene Collection. Probes were synthesized by random priming and Klenow-mediated incorporation of DIG-11-dUTP.

ADDITIONAL FIGURE LEGENDS

Figure S1: SoxN gene expression and binding datasets. (A) Differential GO:BP enrichment of the three groups of genes. Only the 25 most enriched terms for each dataset are shown (See Table S1E, S1G and S1I for the full enrichments). (B) Pairwise comparisons (colour-coded by z-score) between SoxN FDR25 binding datasets. (C) Proportional Venn diagram showing the overlap between the lists of genes assigned to each binding dataset.

Figure S2: Features of SoxN binding. (A) Histogram and probability density showing the minimum distance between SoxN binding intervals and TSSs. (B) Differential GO:BP enrichment for genes assigned to intervals hitting intergenic regions, introns or exons. Only the 25 most enriched terms for each dataset are shown (See Table S2J, S2K and S2L for the full enrichments). (C) Percentage GC content of SoxN binding intervals and surrounding areas. (D) Sequence conservation, expressed as average phastCons score, of the areas surrounding SoxN binding intervals. (E) Top three known motifs overrepresented in the SoxN core dataset. Their best TF match and associated p-value are shown.

Figure S3: SoxN and the chromatin landscape. (A) Pairwise comparisons (colour-coded by z-score) between the SoxN core dataset and binding datasets of TFs from the modENCODE (.M suffix) project. (B) Colour-coded z-scores of pairwise comparisons between the SoxN core dataset, chromatin binding and modifying proteins, histone modifications and TFs datasets from the modENCODE project (.M suffix).

Figure S4: Expression of SoxN direct targets in *SoxN* mutant embryos. Expression patterns of a selection of SoxN direct targets identified in this study in *SoxN* heterozygous

(left) and homozygous embryos (right). With the exception of *gcm*, *ey* and *toy* (*in situ* hybridization), all the images represent immunohistochemistry stainings. (A) Sim, a negative control not showing any expression disruption (B) Proneural genes; (C) NBs temporal identity genes; (D) Genes involved in NBs asymmetric divisions and differentiation, and/or in gliogenesis; (E) Genes involved in different aspects of neural development; (F) Genes involved in the development of neuronal projections. All embryos are shown as ventral views with anterior orientated to the left.

Figure S5: Differences in genes targeted by SoxN and D in wild type embryos. Differential GO:BP enrichment of genes uniquely bound by SoxN, uniquely bound by D or bound by both factors. Only the 25 most enriched terms for each dataset are shown (See Table S5Q, S5R and S5S for the full enrichments).

Figure S6: Changes in SoxN and D binding in wild type, *D* and *SoxN* mutant embryos. GO:BP enrichment of genes associated with the five types of events. (A) Changes in SoxN binding in wild type and *D* mutants. Only the 25 most enriched terms for each dataset are shown (See Table S5D” - S5H” for the full enrichments). (B) Changes in D binding in wild type and *SoxN* mutants. Only the 25 most enriched terms for each dataset are shown (See Table S5I" - S5M" for the full enrichments).

ADDITIONAL TABLE LEGENDS

Table S1: Genes differentially expressed genes in *SoxN* mutants. (A) Probes showing differential expression in *SoxN* mutants, together with their IDs, expression values and p-values. (B) List of differentially expressed genes. (C) GO:BP enrichment of differentially expressed genes. (D) List of downregulated genes. (E) GO:BP enrichment of downregulated genes. (F) List of upregulated genes. (G) GO:BP enrichment of upregulated genes. (H) List of variable genes. (I) GO:BP enrichment of variable genes.

Table S2: The SoxN core dataset. (A) Genomic coordinates of intervals in the core dataset. (B) List of genes assigned to the core dataset. (C) GO:BP enrichment of genes assigned to the core dataset. (D) Genomic coordinates of intervals in the core dataset hitting intergenic regions. (E) Genomic coordinates of intervals in the core dataset hitting introns. (F) Genomic coordinates of intervals in the core dataset hitting exons. (G) List of genes assigned to intervals in the core dataset hitting intergenic regions. (H) List of genes assigned to intervals in the core dataset hitting introns. (I) List of genes assigned to intervals in the core dataset hitting exons. (L) GO:BP enrichment of genes assigned to intervals in the core dataset hitting intergenic regions. (M) GO:BP enrichment of genes assigned to intervals in the core dataset hitting introns. (N) GO:BP enrichment of genes assigned to intervals in the core dataset hitting exons.

Table S3: SoxN direct targets. (A) List of SoxN direct targets. (B) GO:BP enrichment of SoxN direct targets. (C) Gene expression data for direct targets downregulated in *SoxN* mutants. (D) Gene expression data for direct targets upregulated in *SoxN* mutants. (E) Gene expression data for direct targets variably expressed in *SoxN* mutants.

Table S4: Genes bound by SoxN and mouse Sox2 or Sox11. (A) List of *Drosophila*

genes with mouse orthologs bound by Sox2. (B) GO:BP enrichment of SoxN - Sox2 bound genes. (C) List of *Drosophila* genes with mouse orthologs bound by Sox11. (D) GO:BP enrichment of SoxN - Sox11 bound genes. (E) List of *Drosophila* genes with mouse orthologs bound by Sox11 but not Sox2. (F) GO:BP enrichment of SoxN and Sox11 but not Sox2-bound genes.

Table S5: SoxN and D binding in wild type, *D* and *SoxN* mutant embryos. (A) Genomic coordinates of intervals bound by both SoxN and D. (B) Genomic coordinates of intervals uniquely bound by SoxN. (C) Genomic coordinates of intervals uniquely bound by D. (D) Genomic coordinates of intervals bound by SoxN showing no change in wild type and *D* mutant embryos. (E) Genomic coordinates of intervals bound by SoxN showing compensation in *D* mutant embryos. (F) Genomic coordinates of intervals bound by SoxN showing increased binding in *D* mutant embryos. (G) Genomic coordinates of intervals bound by SoxN showing *de novo* binding in *D* mutant embryos. (H) Genomic coordinates of intervals bound by SoxN showing loss of binding in *D* mutant embryos. (I) Genomic coordinates of intervals bound by D showing no change in wild type and *SoxN* mutant embryos. (J) Genomic coordinates of intervals bound by D showing compensation in *SoxN* mutant embryos. (K) Genomic coordinates of intervals bound by D showing increased binding in *SoxN* mutant embryos. (L) Genomic coordinates of intervals bound by D showing *de novo* binding in *SoxN* mutant embryos. (M) Genomic coordinates of intervals bound by D showing loss of binding in *SoxN* mutant embryos. (N) List of genes associated with both SoxN and D binding. (O) List of genes exclusively associated with SoxN binding. (P) List of genes exclusively associated with D binding. (Q) GO:BP enrichment of genes associated with both SoxN and D binding. (R) GO:BP enrichment of genes exclusively associated with SoxN binding. (S) GO:BP enrichment of genes exclusively associated with D binding. (T) List of genes associated with SoxN binding intervals with no change. (U) List

of genes associated with SoxN compensated binding intervals. (V) List of genes associated with intervals displaying SoxN increased binding. (W) List of genes associated with SoxN *de novo* binding intervals. (X) List of genes associated with SoxN loss binding intervals. (Y) List of genes associated with D binding intervals with no change. (Z) List of genes associated with D compensated binding intervals. (A") List of genes associated with intervals displaying D increased binding. (B”) List of genes associated with D *de novo* binding intervals. (C") List of genes associated with D loss binding intervals. (D”) GO:BP enrichment of genes associated with SoxN binding intervals with no change. (E”) GO:BP enrichment of genes associated with SoxN compensated binding intervals. (F”) GO:BP enrichment of genes associated with intervals displaying SoxN increased binding. (G”) GO:BP enrichment of genes associated with SoxN *de novo* binding intervals. (H”) GO:BP enrichment of genes associated with SoxN loss binding intervals. (I") GO:BP enrichment of genes associated with D binding intervals with no change. (J") GO:BP enrichment of genes associated with D compensated binding intervals. (K") GO:BP enrichment of genes associated with intervals displaying D increased binding. (L") GO:BP enrichment of genes associated with D *de novo* binding intervals. (M") GO:BP enrichment of genes associated with D loss binding intervals.

Table S6: SoxN and D binding intervals targeting FlyLight enhancers with reported CNS expression. (A) Genomic coordinates of FlyLight enhancers targeted by both SoxN and D. (B) Genomic coordinates of FlyLight enhancers targeted uniquely by SoxN. (C) Genomic coordinates of FlyLight enhancers targeted only by D.

ADDITIONAL REFERENCES

1. Karess RE: **P element mediated germ line transformation of Drosophila**. In *DNA Cloning Vol II*. Edited by Glover DM. IRL Press; 1985:121–142.

2. Ghanim M, White KP: **Genotyping method to screen individual Drosophila embryos prior to RNA extraction.** *Biotechniques* 2006, **41**:414, 416, 418.

3. Vogel MJ, Peric-Hupkes D, van Steensel B: **Detection of in vivo protein-DNA interactions using DamID in mammalian cells.** *Nat Protoc* 2007, **2**:1467–78.

4. Sandmann T, Jakobsen JS, Furlong EEM: **ChIP-on-chip protocol for genome-wide analysis of transcription factor binding in Drosophila melanogaster embryos.** *Nat Protoc* 2006, **1**:2839–55.

5. Buhler J, Ideker T, Haynor D: **Dapple: improved techniques for finding spots on DNA microarrays**. *Univ Washingt CSE Tech Rep UWTR* 2000:08–05.

6. Huber W, von Heydebreck A, Sültmann H, Poustka A, Vingron M: **Variance stabilization applied to microarray data calibration and to the quantification of differential expression.** *Bioinformatics* 2002, **18 Suppl 1**:S96–104.

7. Smyth G: **Limma: linear models for microarray data**. In *Bioinforma Comput Biol Solut Using R Bioconductor*. Springer; 2005:397–420.

8. Toedling J, Skylar O, Sklyar O, Krueger T, Fischer JJ, Sperling S, Huber W: **Ringo--an R/Bioconductor package for analyzing ChIP-chip readouts.** *BMC Bioinformatics* 2007, **8**:221.

9. Nicol JW, Helt GA, Blanchard SG, Raja A, Loraine AE: **The Integrated Genome Browser: free software for distribution and exploration of genome-scale datasets.** *Bioinformatics* 2009, **25**:2730–1.

10. Fischer B, Ferrero E, Stojnic R, Russell S: **SimBindProfiles: a Bioconductor package to identify common and unique regions in genome tiling array data**. 2013.

11. Quinlan AR, Hall IM: **BEDTools: a flexible suite of utilities for comparing genomic features.** *Bioinformatics* 2010, **26**:841–2.

12. Maere S, Heymans K, Kuiper M: **BiNGO: a Cytoscape plugin to assess overrepresentation of gene ontology categories in biological networks.** *Bioinformatics* 2005, **21**:3448–9.

13. Heinz S, Benner C, Spann N, Bertolino E, Lin YC, Laslo P, Cheng JX, Murre C, Singh H, Glass CK: **Simple combinations of lineage-determining transcription factors prime cis-regulatory elements required for macrophage and B cell identities.** *Mol Cell* 2010, **38**:576–89.

14. Grant CE, Bailey TL, Noble WS: **FIMO: scanning for occurrences of a given motif.** *Bioinformatics* 2011, **27**:1017–8.

15. Bickel PJ, Boley N, Brown JB, Huang H, Zhang NR: **Subsampling methods for genomic inference**. *Ann Appl Stat* 2010, **4**:1660–1697.

16. Nègre N, Brown CD, Ma L, Bristow CA, Miller SW, Wagner U, Kheradpour P, Eaton ML, Loriaux P, Sealfon R, Li Z, Ishii H, Spokony RF, Chen J, Hwang L, Cheng C, Auburn RP, Davis MB, Domanus M, Shah PK, Morrison C a., Zieba J, Suchy S, Senderowicz L, Victorsen A, Bild N a., Grundstad a. J, Hanley D, MacAlpine DM, Mannervik M, et al.: **A cis-regulatory map of the Drosophila genome.** *Nature* 2011, **471**:527–31.

17. Li X, MacArthur S, Bourgon R, Nix D, Pollard DA, Iyer VN, Hechmer A, Simirenko L,

Stapleton M, Luengo Hendriks CL, Chu HC, Ogawa N, Inwood W, Sementchenko V, Beaton A, Weiszmann R, Celniker SE, Knowles DW, Gingeras T, Speed TP, Eisen MB, Biggin MD: **Transcription factors bind thousands of active and inactive regions in the Drosophila blastoderm.** *PLoS Biol* 2008, **6**:e27.

18. Kharchenko P V, Alekseyenko AA, Schwartz YB, Minoda A, Riddle NC, Ernst J, Sabo PJ, Larschan E, Gorchakov AA, Gu T, Linder-Basso D, Plachetka A, Shanower G, Tolstorukov MY, Luquette LJ, Xi R, Jung YL, Park RW, Bishop EP, Canfield TK, Sandstrom R, Thurman RE, MacAlpine DM, Stamatoyannopoulos JA, Kellis M, Elgin SCR, Kuroda MI, Pirrotta V, Karpen GH, Park PJ: **Comprehensive analysis of the chromatin landscape in Drosophila melanogaster.** *Nature* 2011, **471**:480–5.

19. Kumar S, Konikoff C, Van Emden B, Busick C, Davis KT, Ji S, Wu L-W, Ramos H, Brody T, Panchanathan S, Ye J, Karr TL, Gerold K, McCutchan M, Newfeld SJ: **FlyExpress: visual mining of spatiotemporal patterns for genes and publications in Drosophila embryogenesis.** *Bioinformatics* 2011, **27**:3319–20.

20. Murali T, Pacifico S, Yu J, Guest S, Roberts GG, Finley RL: **DroID 2011: a comprehensive, integrated resource for protein, transcription factor, RNA and gene interactions for Drosophila.** *Nucleic Acids Res* 2011, **39**(Database issue):D736–43.

21. Smoot ME, Ono K, Ruscheinski J, Wang P-L, Ideker T: **Cytoscape 2.8: new features for data integration and network visualization.** *Bioinformatics* 2011, **27**:431–2.

22. Patel NH, Goldstein LSB, Fyrberg EA: **Drosophila melanogaster: Practical uses in cell and molecular biology**. In *San Diego Acad*. Academic Press; 1994:445–487.

23. Skeath JB, Carroll SB: **Regulation of achaete-scute gene expression and sensory organ pattern formation in the Drosophila wing.** *Genes Dev* 1991, **5**:984–95.

24. Condie JM, Mustard JA, Brower DL: **Generation of anti-Antennapedia monoclonal antibodies and Antennapedia protein expression in imaginal discs.** *Drosoph Inf Serv* 1991, **70**:52–4.

25. Weng M, Golden KL, Lee C-Y: **dFezf/Earmuff maintains the restricted developmental potential of intermediate neural progenitors in Drosophila.** *Dev Cell* 2010, **18**:126–35.

26. Kambadur R, Koizumi K, Stivers C, Nagle J, Poole SJ, Odenwald WF: **Regulation of POU genes by castor and hunchback establishes layered compartments in the Drosophila CNS.** *Genes Dev* 1998, **12**:246–60.

27. Blochlinger K, Bodmer R, Jan LY, Jan YN: **Patterns of expression of cut, a protein required for external sensory organ development in wild-type and cut mutant Drosophila embryos.** *Genes Dev* 1990, **4**:1322–31.

28. Soriano NS, Russell S: **The Drosophila SOX-domain protein Dichaete is required for the development of the central nervous system midline.** *Development* 1998, **125**:3989–96.

29. Lacin H, Zhu Y, Wilson BA, Skeath JB: **dbx mediates neuronal specification and differentiation through cross-repressive, lineage-specific interactions with eve and hb9.** *Development* 2009, **136**:3257–66.

30. Isshiki T, Pearson B, Holbrook S, Doe CQ: **Drosophila neuroblasts sequentially express transcription factors which specify the temporal identity of their neuronal progeny.** *Cell* 2001, **106**:511–21.

31. MacArthur S, Li X-Y, Li J, Brown JB, Chu HC, Zeng L, Grondona BP, Hechmer A, Simirenko L, Keränen SVE, Knowles DW, Stapleton M, Bickel P, Biggin MD, Eisen MB:

Developmental roles of 21 Drosophila transcription factors are determined by quantitative differences in binding to an overlapping set of thousands of genomic regions. *Genome Biol* 2009, **10**:R80.

32. Cai Y, Chia W, Yang X: **A family of snail-related zinc finger proteins regulates two distinct and parallel mechanisms that mediate Drosophila neuroblast asymmetric divisions.** *EMBO J* 2001, **20**:1704–14.

33. Jinushi-Nakao S, Arvind R, Amikura R, Kinameri E, Liu AW, Moore AW: **Knot/Collier and cut control different aspects of dendrite cytoskeleton and synergize to define final arbor shape.** *Neuron* 2007, **56**:963–78.

34. Kosman D, Small S, Reinitz J: **Rapid preparation of a panel of polyclonal antibodies to Drosophila segmentation proteins.** *Dev Genes Evol* 1998, **208**:290–4.

35. Stagg SB, Guardiola AR, Crews ST: **Dual role for Drosophila lethal of scute in CNS midline precursor formation and dopaminergic neuron and motoneuron cell fate.** *Development* 2011, **138**:2171–83.

36. Giniger E, Tietje K, Jan LY, Jan YN: **lola encodes a putative transcription factor required for axon growth and guidance in Drosophila.** *Development* 1994, **120**:1385–98.

37. Leal SM, Qian L, Lacin H, Bodmer R, Skeath JB: **Neuromancer1 and Neuromancer2 regulate cell fate specification in the developing embryonic CNS of Drosophila melanogaster.** *Dev Biol* 2009, **325**:138–50.

38. Kuzin A, Brody T, Moore AW, Odenwald WF: **Nerfin-1 is required for early axon guidance decisions in the developing Drosophila CNS.** *Dev Biol* 2005, **277**:347–65.

39. Yeo SL, Lloyd A, Kozak K, Dinh A, Dick T, Yang X, Sakonju S, Chia W: **On the functional overlap between two Drosophila POU homeo domain genes and the cell fate specification of a CNS neural precursor.** *Genes Dev* 1995, **9**:1223–36.

40. Grosskortenhaus R, Robinson KJ, Doe CQ: **Pdm and Castor specify late-born motor neuron identity in the NB7-1 lineage.** *Genes Dev* 2006, **20**:2618–27.

41. Hirth F, Kammermeier L, Frei E, Walldorf U, Noll M, Reichert H: **An urbilaterian origin of the tripartite brain: developmental genetic insights from Drosophila.** *Development* 2003, **130**:2365–73.

42. Spana EP, Doe CQ: **The prospero transcription factor is asymmetrically localized to the cell cortex during neuroblast mitosis in Drosophila.** *Development* 1995, **121**:3187–95.

43. Yu HH, Araj HH, Ralls SA, Kolodkin AL: **The transmembrane Semaphorin Sema I is required in Drosophila for embryonic motor and CNS axon guidance.** *Neuron* 1998, **20**:207–20.

44. O'Connor-Giles KM, Skeath JB: **Numb inhibits membrane localization of Sanpodo, a four-pass transmembrane protein, to promote asymmetric divisions in Drosophila.** *Dev Cell* 2003, **5**:231–43.

45. Tautz D, Pfeifle C: **A non-radioactive in situ hybridization method for the localization of specific RNAs in Drosophila embryos reveals translational control of the segmentation gene hunchback.** *Chromosoma* 1989, **98**:81–5.
